# Ultra‐Low‐Cost Hydrophobic Organic Coating for Highly Reversible Zinc Anodes

**DOI:** 10.1002/anie.202523567

**Published:** 2026-02-03

**Authors:** Shixun Wang, Zhiquan Wei, Yiqiao Wang, Shengnan Wang, Dedi Li, Hu Hong, Chuan Li, Yanbo Wang, Zhuoxi Wu, Shaoce Zhang, Xueying Zheng, Yi‐Chun Lu, Chunyi Zhi

**Affiliations:** ^1^ Department of Mechanical Engineering The University of Hong Kong Hong Kong, S.A.R. P. R. China; ^2^ Department of Materials Science and Engineering City University of Hong Kong Hong Kong, S.A.R. P. R. China; ^3^ Electrochemical Energy and Interfaces Laboratory, Department of Mechanical and Automation Engineering The Chinese University of Hong Kong Hong Kong, S.A.R. China; ^4^ Materials Innovation Institute For Life Sciences and Energy (MILES) HKU‐SIRI Shenzhen China; ^5^ Center For Energy Storage The University of Hong Kong Hong Kong, Pokfulam China

**Keywords:** additives, aqueous zinc batteries, dendrite growth, hydrophobic organics

## Abstract

Electrolyte additive engineering offers a promising pathway for achieving dendrite‐free aqueous zinc‐ion batteries (ZIBs) while facing challenges related to hydrophilic characteristics and/or high loading requirements. Herein, we developed a cost‐effective and scalable facile immersion treatment to deposit a hydrophobic 1,3‐Di(o‐tolyl)thiourea (DTH) layer with nanoscale thickness (≤ 14 nm). This approach yields an ultra‐low DTH loading (5.37 × 10^−13^ M) and exceptional cost efficiency (1.43 × 10^−7^ USD Ah^−1^), surpassing conventional water‐miscible organic additives and biomass‐derived counterparts by orders of magnitude. The hydrophobic DTH layer optimizes Zn electrochemistry and mitigates parasitic reactions, irrespective of the immersion sequence in the same batch of ethanol solution. Consequently, the Zn||ODASnI_4_ (ODA denotes 1,8‐octadiamine) coin cell demonstrated stable operation over 2500 cycles at 2 A g^−1^ with a low additive cost of 1.43 × 10^−6^ USD per cell. The pouch cell showed an average coulombic efficiency (CE) of 99.9% and 72% capacity retention after 1200 cycles, incurring an ultra‐low additive cost of 7.02× 10^−5^ USD while delivering a high energy density of 143 Wh kg^−1^ (based on cathode mass). This work enabled durable and high‐performance ZIBs at minimal cost, providing a foundation for further exploration of low‐cost, scalable strategies in aqueous battery systems.

## Graphical abstract

A nanoscale hydrophobic 1,3‐Di(o‐tolyl)thiourea (DTH) layer (<14 nm) was deposited on zinc anodes for durable aqueous Zn‐ion batteries, costing merely 1.43×10^−7^ USD·Ah^−1^. By suppressing side reactions and dendrites, it achieved 2500‐cycle coin cells and a 143 Wh·kg^−1^ pouch cell (1200 cycles) with a record‐low additive cost (7.02×10^−7^ USD).

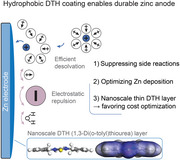

## 1 Introduction

Zinc metal, renowned for its high natural abundance, cost‐effectiveness, low redox potential (−0.76 V vs. standard hydrogen electrode, SHE), and impressive energy density (820 mAh g^−1^), stands out as one of the most promising anode materials for rechargeable aqueous batteries [1, 2, 3, 4]. Yet, the intrinsic properties of zinc metal in mild acidic aqueous electrolytes, such as inducing parasitic hydrogen evolution (HER) and the nucleation of zinc hydroxyl byproducts, contribute to the excessive consumption of electrolytes and the proliferation of zinc dendrites that ultimately lead to battery failure [5, 6, 7, 8, 9]. The associated pH perturbation during Zn deposition further leads to the dissolution of cathode materials like vanadium oxides, resulting in irreversible capacity loss [10].

Current strategies in surface and additive engineering often overlook cost considerations, despite their effectiveness in optimizing zinc chemistry. Surface engineering, in particular, involves intricate alloy designs [11] and the use of sophisticated composites [12], such as metal‐organic frameworks [13], biomimetic materials [14], and mixtures of metal oxides with carbon materials [15], which not only complicate the process but also drive up cost. Additive engineering, on the other hand, has garnered substantial attention for its cost‐effectiveness, contrasting with conventional approaches that necessitate resource‐intensive processes like meticulous geometric tailoring of zinc or separators.[16, 17, 18, 19, 20, 21, 22] Still, the economic viability of the additive strategy has long been overlooked, concerning challenges from high feed dosages and/or reliance on expensive functional additives.[23, 24] Those electrolyte additives ranging from water‐miscible organics to molecules with electrophilic/nucleophilic functional groups are commonly tailored to modulate the solvation structure of Zn^2+^ ions and/or disrupt intermolecular hydrogen bonds in water, thereby directing ion flux toward improving kinetics, suppressing parasitic reactions, and reversible Zn plating/stripping [25, 26, 27, 28]. However, the required additive concentrations (1 ∼ 3 M) to displace solvated water molecules in the electrolytes often rival or exceed those of the electrolyte, significantly escalating the overall electrolyte expense. For instance, dimethyl sulfoxide (DMSO) can weaken the bond strength between Zn^2+^ and H_2_O in the Zn^2+^ solvation structure, while an effective ZnCl_2_‐H_2_O‐DMSO electrolyte requires 20 wt% DMSO (2.5 M), exceeding the ZnCl_2_ electrolyte concentration (1 M) by nearly threefold [25]. The overlooked cost issue is further exacerbated by the reliance on high‐value additives such as graphene oxide (27 USD g^−1^), sericin (223 USD g^−1^), and biomass hyaluronic acid (2392 USD g^−1^) 29, 30, 31], regardless of their proven efficacy in regulating Zn electrochemistry.

Nevertheless, the reliance on water‐soluble additives narrows the scope for effective hydrophobic options and may introduce additional complications for the cathode electrode. For instance, water‐miscible organic additives featuring strong electron‐donating ability can cause severe dissolution of conversion‐type materials (such as halogen and chalcogen elements) in aqueous electrolytes [32, 33, 34]. Constructing a nanoscale hydrophobic functional layer between the zinc anode and the aqueous electrolyte offers a solid interface to mediate the differences in electrode materials and enhance overall battery reversibility. However, in practice, most electrolyte additives remain polar and hydrophilic, with no successful development of hydrophobic organic alternatives reported thus far. Even for dense solid electrolyte interfaces (SEI), their complicated fabrication procedure, dissolution and/or decomposition of SEI, and limited effectiveness create new challenges for practical applications.[35, 36, 37, 38, 39] These issues underscore the need for innovative solutions to advancing hydrophobic organics for dendrite‐free zinc ion batteries.

Herein, we propose to load hydrophobic 1,3‐Di(o‐tolyl)thiourea (DTH) with nanoscale thickness onto the surface of the zinc anode, aiming to optimize Zn deposition and mitigate parasitic reactions with water, such as the HER and the formation of insulating basic zinc sulfates. Though DTH shares a similar tendency to accumulate on the zinc surface with commonly used water‐soluble reagents, it barely dissolves in aqueous solutions and can quickly reach saturation in ethanol. This characteristic facilitates a convenient (5‐s immersion treatment) and cost‐effective (1.43 × 10^−7^ USD Ah^−1^) surface treatment approach for zinc anodes (hereafter denoted as DTH@Zn). The hydrophobic DTH layer could effectively regulate the electrical double layer and Zn stripping/plating. As a result, the DTH@Zn||Cu asymmetric cell sustained long‐term operation of 2000 h at 10 mA cm^−2^, 1 mAh cm^−2^ with a high average CE of 99.87%. The DTH@Zn||DTH@Zn symmetric cell experienced over 2000 h at 10 mA cm^−2^, 4 mAh cm^−2^, with a crystallographic deviation factor (*f_D_*) down to 0.8. The solid zinc chemistry arising from the DTH layer further upholds the steady operation of DTH@Zn||ODASnI_4_ (ODA denotes 1,8‐octadiamine) coin and pouch cells that sustained 2500 cycles at 2 A g^−1^ and 1200 cycles at 3.3 mA cm^−2^, with an average CE of around 99.6% and 99.9%, respectively.

## 2 Results and Discussion

### 2.1 Working Principle of DTH on Zinc Surface

**FIGURE 1:**
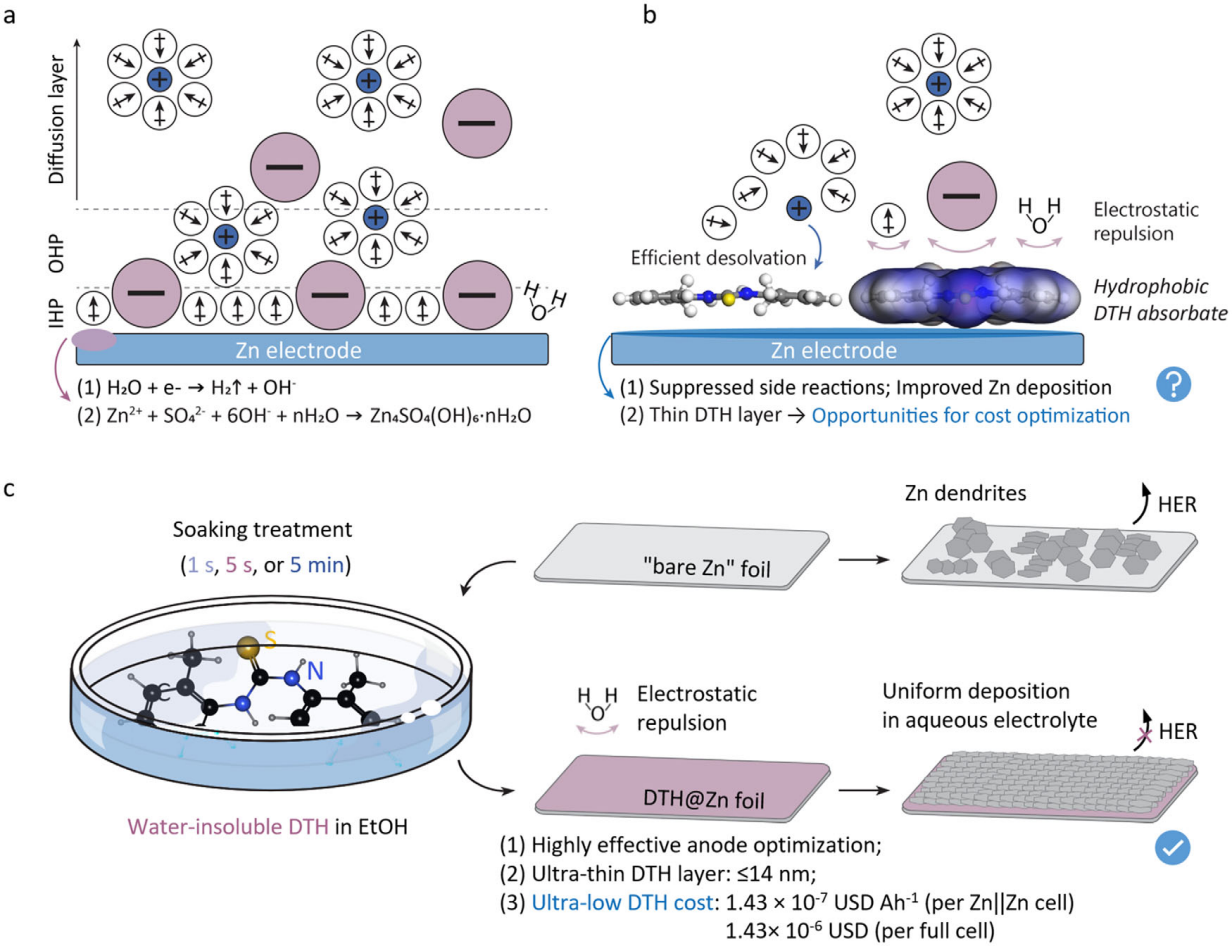
Potential performance of hydrophobic DTH layer on zinc surface and associated preparation method. Helmholtz plane near **a** “bare zinc” electrode and **b** that coupled with DTH layer in ZnSO_4_ electrolyte; **c** The simplified process of loading DTH layer onto zinc electrode with associated benefits. The dosage of the electrolyte in half‐ and full‐cells was 80 µL. The DTH cost was derived from Zn||Zn symmetric cells and Zn||ODASnI_4_ full cells, as will be discussed later.

In an electric conductor, the interruption of its high‐periodic crystal lattice leads to the presence of dangling bonds on the surface. These undercoordinated atoms create a surface with a strong affinity for attracting counterions, such as anions and polar molecules, from the electrolyte, resulting in the formation of an inner Helmholtz plane (IHP) and an outer Helmholtz plane (OHP) that loosely associates with the conductor surface. However, this configuration inevitably gives rise to the decomposition of water molecules near the zinc anode, resulting in an increase in local pH value that obliges the nucleation of insulating basic zinc sulfate hydrates, Zn_4_SO_4_(OH)_6_∙nH_2_O, in the widely used ZnSO_4_ electrolyte (Figure 1a) [30, 40, 41, 42]. As depicted in Figure 1b, the deposition of a hydrophobic DTH layer as SEI is expected to modify the electrical double layer in such a way that water molecules are repelled from the zinc surface to facilitate the desolvation process, contributing to a significant reduction in side effects, including HER and dendrite growth, and offering opportunities for cost optimization that has long been ignored.

**FIGURE 2:**
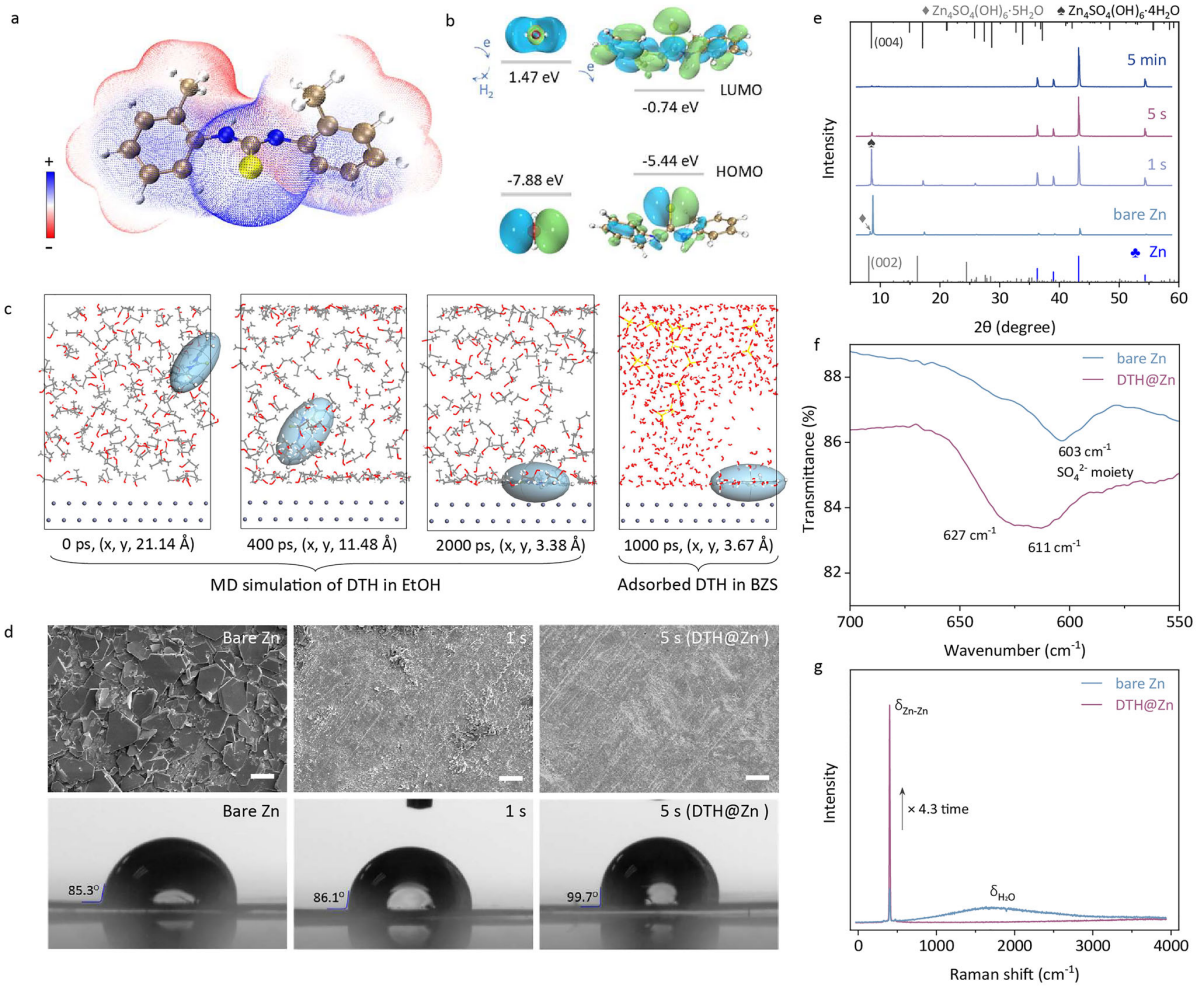
Theoretical calculation and function of DTH layer. **a** Molecular electrostatic potential map of DTH; **b** HOMO‐LUMO energy diagram of H_2_O (left) and DTH (right); **c** MD simulation of DTH in EtOH and BZS (ZnSO_4_ electrolyte); **d** SEM and contact angle images; **e** XRD patterns of “bare Zn” and DTH‐treated Zn plates after immersion in BZS for 1 week; **f** FTIR and **g** Raman spectra of the above‐mentioned “bare Zn” and DTH@Zn. In panel **a–c**, the color scheme for the spheres is defined as follows: brown is carbon, blue is nitrogen, red is oxygen, white is hydrogen, light grey is zinc, and yellow represents sulfur atoms. The light blue oval in panel **c** highlights the spatial location of DTH during MD simulations. The bar size in panel d is 20 µm.

To validate the ground‐up design, we submerged the polished zinc electrode in a DTH ethanol (EtOH) solution with different treatment durations (1 s, 5 s, or 5 min), aiming to realize an in‐situ deposition of a hydrophobic DTH layer on the zinc surface with a varying thickness (Figure 1c). The visualized charge distribution of the DTH molecule in Figure 2a highlights the negatively charged regions surrounding the C = S and phenyl groups, which promote high adsorption energy of −3.26 eV and −2.93 eV on the (002) and (101) lattice plane of zinc metal, respectively (Figure S1). In addition, DTH possesses a significantly lower lowest unoccupied molecular orbital (LUMO) energy level than H_2_O (Figure 2b). This difference theoretically creates preferred electron channels to suppress undesired HER. Molecular dynamics (MD) simulations in Figure 2c provide further insights into the behavior of hydrophobic DTH molecules in EtOH after immersing the zinc anode. The simulation shows that DTH molecules can rapidly attach to the zinc surface within 2000 ps, reaching a minimum system energy (Figure S2). Even after transferring to a 2 M ZnSO_4_ electrolyte (referred to as BZS), the adsorption of DTH on the zinc surface remains stable in IHP so as to manipulate the deposition of Zn (Figure S3). Moreover, the DTH molecules exhibit high mobility on the zinc metal surface, with a remarkably low migration energy of 0.02 eV on Zn (002) and 0.09 eV on Zn (101), potentially favoring DTH coating efficiency and facilitating zinc flux regulation (Figure S4). The DTH accumulation can modulate the mobility of Zn^2+^ ions and alter their first solvation shell, thereby facilitating the desolvation process and ensuring more uniform Zn deposition (Figure S5). Atomic force microscopy (AFM) images in Figure S6 show a smoother zinc surface following the deposition of the hydrophobic DTH layer, indicating reduced zinc corrosion during storage under high‐humidity conditions (>80% RH, 25 °C).

The scanning electron microscope (SEM) images in Figure 2d and the confocal optical microscopy photographs in Figure S7 clearly depict a noticeable accumulation of basic zinc sulfate, indicating the occurrence of HER on the surface of “bare zinc” after being immersed in BZS for 7 days. In contrast, these byproducts are nearly absent when a nanoscale‐thick DTH layer is deposited through a 5‐second immersion treatment (denoted as DTH@Zn unless otherwise specified), consistent with the remarkably restrained signals from oxygen (Figure S8). This DTH layer improves the hydrophobic characteristics of the coated zinc anode, effectively reducing the occurrence of spontaneous zinc corrosion in BZS (Figure S9). The x‐ray diffraction (XRD) patterns in Figure 2e and Figure S10 further reveal the suppressed byproduct nucleation (originating from HER) on the zinc plate as the immersion period increases from 1 s to 5 s and 5 min. The presence of basic zinc sulfate hydrates, Zn_4_SO_4_(OH)_6_∙nH_2_O (n = 4, or 5), diminished rapidly as the DTH treatment time reached 5 s. The absence of extra crystalline diffraction peaks also suggests that DTH probably forms an amorphous molecular layer coating the zinc surface, as only metallic zinc signatures are observed. Fourier transform infrared spectroscopy (FTIR) spectra in Figure 2f and Figure S11 present the blue‐shifted signals from the SO_4_2^−^ moiety and the O‐H bond after DTH deposition, resulting from decreased mass of the corresponding molecules [43]. Raman spectra in Figure 2g further reveal enhanced Zn‐Zn vibration from the surface of DTH@Zn without signals related to hydrated water, convincing the suppressed corrosion of zinc in BZS.

### 2.2 Plating/Stripping Reversibility

**FIGURE 3:**
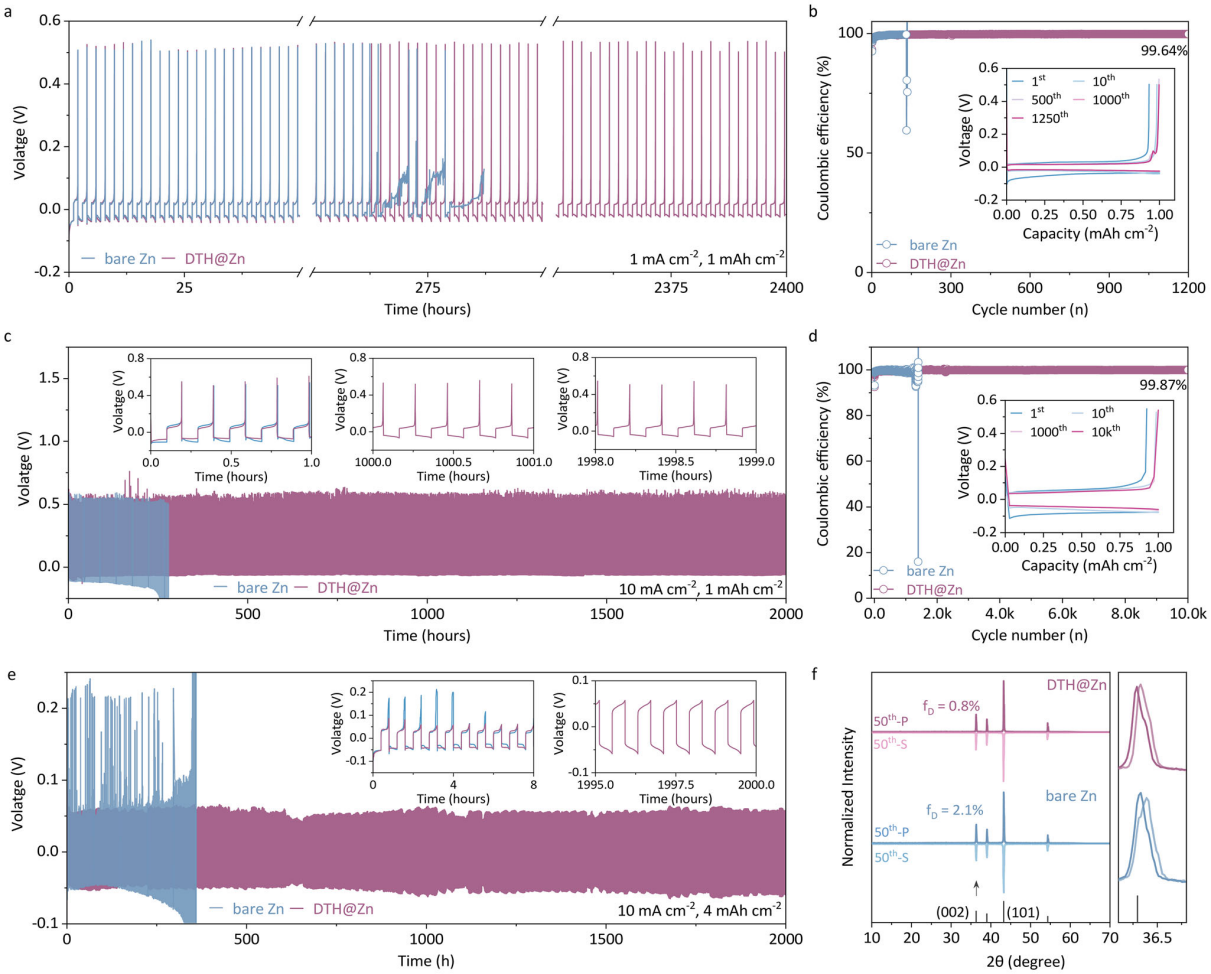
Reversibility of Zn deposition on DTH@Zn in half cells. **a** Galvanostatic cycling of Zn||Cu asymmetric cells at 1 mA cm^−2^, 1 mAh cm^−2^ and **b** the corresponding CEs; **c** Galvanostatic cycling of Zn||Zn symmetric cells at 10 mA cm^−2^, 1 mAh cm^−2^ and **d** the corresponding CEs; **e** Galvanostatic cycling of Zn||Zn symmetric cells at 10 mA cm^−2^, 4 mAh cm^−2^; **f** XRD patterns of stripped/plated “bare Zn” and DTH@Zn electrodes. Inset in **b**, **d** are the corresponding voltage profiles at different cycles; Inset in **c**, **e** are selected cycling slices. The frame in the right of panel f is an enlargement.

The advantages of surface modification were further evaluated in the form of half‐cells. As shown in Figure 3a,b, the DTH@Zn||Cu asymmetric cell facilitated 2400 h of operation at 1 mA cm^−2^, 1 mAh cm^−2^ with a high average CE of 99.6%, whereas the pristine “bare Zn”||Cu cell failed in 150 cycles (< 300 h). Meanwhile, the DTH layer enabled a slightly larger voltage polarization (39.7 meV) than that of the pristine cell (38.5 meV) in the initial phase of the cycling process, suggesting relatively increased nucleation energy as the inclusion of the DTH layer (Figure S12). Under harsh cycling conditions, the DTH@Zn||Cu configuration endured 2000 h (10,000 cycles) at 10 mA cm^−2^, 1 mAh cm^−2^ with a high average CE of 99.0% (Figure 3c,d). Instead, the “bare Zn” | Cu cell suffered from side reactions and Zn dendrite formation, leading to increasing voltage polarization, a decline in CE, and eventual failure within 300 h. As expected, the DTH@Zn||DTH@Zn symmetric cell demonstrated stable and reversible cycling at 10 mA cm^−2^ and 4 mAh cm^−2^ for 2000 h, with no signs of voltage polarization and (soft) short circuits (Figure 3e). The plated zinc anode underwent additional evaluation using confocal optical microscopy, as depicted in Figure S13. Instead, DTH@Zn exhibited a clean morphology, contrasting with the byproduct‐rich surface of the “bare Zn” sample. Raman measurements in Figure S14 highlighted the persistent presence of signals from water on the surface of “bare Zn”, a phenomenon absent on the cycled DTH@Zn sample.

We further inspected the mathematical correlation of Zn deposition on the two symmetric Zn electrodes after 50 cycles at 1 mA cm^−2^, 1 mAh cm^−2^, according to the equation *f*_D_ = |*I*_P(0 0 2)_/*I*_P(1 0 1)_ −*I*_S(0 0 2)_/*I*_S(1 0 1)_|  × 100% where *I*_P_ and *I*_S_ are the diffraction intensity of the plated and stripped side, respectively [44]. As shown in Figure 3f, the pristine sample exhibited an *f*_D_ value of 2.1% while DTH@Zn||DTH@Zn showed a smaller deviation factor of 0.8%, nearly one‐third of that observed in “bare Zn”||“bare Zn”. In contrast to the control sample, which displayed an abundance of zinc dendrites and byproducts, the stripped and plated zinc electrodes with the DTH layer presented a smooth surface morphology, as illustrated in the SEM images in Figure S15.

### 2.3 Electrochemical Properties and Effectiveness

**FIGURE 4:**
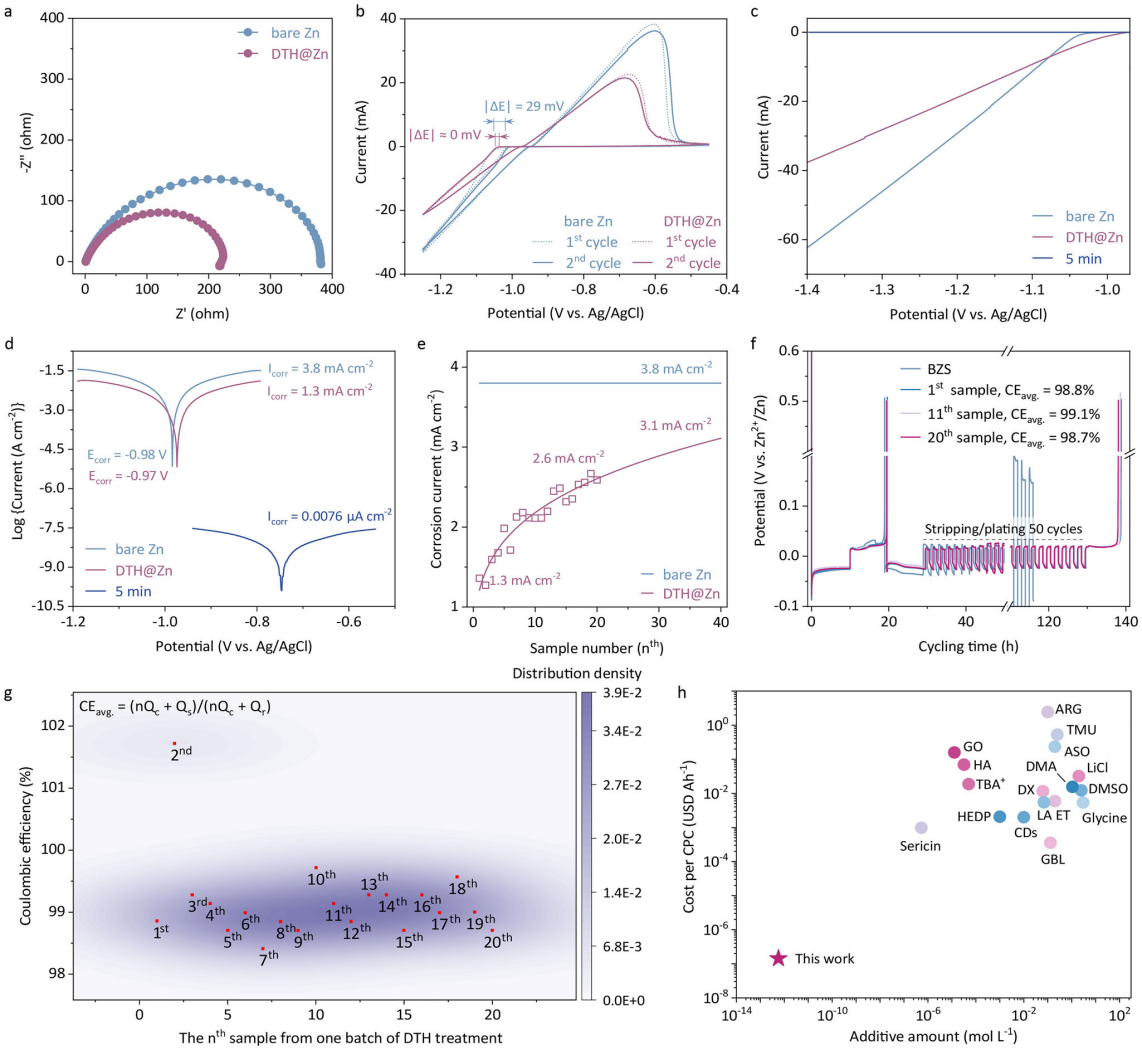
Electrochemical properties of DTH@Zn electrode. **a** EIS spectra, **b** CV curves, **c** LSV curves, and **d** linear polarization curves of Zn||Ti cells based on “bare Zn” and “DTH@Zn”, respectively; **e** Evolution of the corrosion current of twenty Zn plates from the same batch of DTH treatment; **f** “Reservoir” protocol for evaluating average Zn plating/stripping CE and **g** the distribution of those twenty Zn plates' CE; **h** Cost analysis of cumulative plated capacity (CPC) and the feeding amount of DTH and other reported additives. Note that ‘DTH@Zn’ denotes a 5‐second immersion treatment in the DTH ethanol solution, while ‘5 min’ refers to the same treatment but extended to 5 min. The distribution density in **h** is generated by constructing contour lines as functions of two variables: the sample ID and its corresponding CE. The highest density region is represented by the dark violet area in the contour plot. The formula in panel **g** is for the calculation of average coulombic efficiency (CE_avg._), in which *Q_r_* is the capacity of the Zn reservoir, *Q_s_* is the capacity generated during a complete stripping process, and *Q_c_* is a fixed capacity of Zn that was cycled *n* times. The abbreviation in panel **h** is: GO, Graphene oxide [29]; HA, Hyaluronic acid [31]; TBA^+^, Tetrabutylammonium sulfate [49]; LiCl, lithium chloride [50]; DX, 1,3‐Dioxane [55]; GBL, γ‐Butyrolactone [53]; ET, Ectoine [54]; ARG, arginine; TMU, Tetramethylurea; (27) ASO, Al_2_(SO_4_)_3_; (57) LA, Lactobionic acid; (51) DMSO, dimethyl sulfoxide; (25) CDs, cyclodextrins; (56) HEDP, 1‐hydroxy ethylidene‐1,1‐diphosphonic acid; (52) DMA, N,N‐Dimethylacetamide. (26.)

The electrochemical impedance spectroscopy (EIS) spectra in Figure 4a showcase the nearly halved charge transfer resistance after DTH treatment, supported by the derived distribution of relaxation times (DRT) analysis in Figure S16 [45, 46, 47]. Cyclic voltammetry (CV) curves of the “bare Zn”||Ti cell deliver a more significant potential polarization and much higher current than that of DTH@Zn||Ti, arising from sluggish interfacial ion transport and parasitic HER (Figure 4b). Furthermore, the DTH@Zn||Ti cell exhibited a constant nucleation voltage over the twenty cycles, together with a highly reversible plating/stripping process (Figure S17). In contrast, the control sample experienced a rapid voltage drop of 29 mV in the first two cycles due to uneven zinc plating. The linear sweep voltammetry (LSV) curves in Figure 4c further verified the effectiveness of the DTH layer in suppressing side reactions, and importantly, revealed a slightly reduced onset potential compared to “bare Zn”||Ti. This lower onset potential, indicative of a decreased nucleation overpotential, parallels the suppression of voltage polarization of the DTH@Zn||Cu cell observed in Figure 3c. It is worth noting that the zinc surface became insulated after a 5‐minute DTH treatment, as indicated by the near‐zero current observed. Similarly, the linear polarization curves in Figure 4d reveal a negligible corrosion current when excessive DTH processing (5‐minute immersion) was applied, indicating that the thick DTH film effectively could block ion/charge transport. In comparison, the corrosion current of the DTH@Zn||Ti cell was 1.3 mA cm^−2^, approximately one‐third of “bare Zn”||“bare Zn”. The observations emphasize improved electrochemical performance and corrosion resistance on the zinc electrode with specific DTH thickness.

In order to assess the efficacy of the immersion treatment, we prepared 20 zinc plates (0.49 cm^−2^) for successive treatment in a 20 mL EtOH solution containing 10 mg of DTH. As shown in Figure 4e, the corrosion current increased progressively from 1.3 mA cm^−2^ for the first sample to 2.6 mA cm^−2^ for the 20^th^ sample, with a projected value of 3.1 mA cm^−2^ for the 40^th^ sample. Despite this rise, the current density remained substantially lower than that of the control sample (3.8 mA cm^−2^), indicating that the DTH coating effectively suppressed zinc corrosion and side reactions (Figure S18). The beneficial DTH layer is further confirmed by the “reservoir‐half‐cell” galvanostatic protocol as shown in Figure 4f [48]. The control sample, as expected, experienced severe HER issues embodied in the sudden voltage polarization (open circuit) during the test. Irrespective of the treatment sequence, the first, 11^th^, and 20^th^ treated samples exhibited favorable reversible zinc stripping/plating in Zn||Cu asymmetric cells with an average CE of over 98.7%. This feature (CE generally close to 99%) observed across the 20 zinc plates was depicted in Figure 4g, verifying the consistent and dependable enhancement of zinc chemistry through the DTH treatment. A cross‐sectional comparison revealed the high affordability of DTH treatment, particularly in terms of feeding dosage of additive and cost per cumulative plated capacity (CPC), as summarized in Figure 4h and Table S1. As detailed in Note S1, this immersion treatment demonstrates remarkably low DTH consumption, equivalent to a DTH concentration of 5.37 × 10^−13^ M, calculated for an electrolyte addition of 80 µL per cell, significantly lower than the additive amount reported in prior literature [25, 26, 27, 29, 30, 31, 40, 41, 49, 50, 51, 52, 53, 54, 55, 56, 57]. Beyond cost benefits, the DTH treatment also improves the electrochemical performance of Zn||Zn symmetric cell, with an ultra‐low CPC of 1.43 × 10^−7^ USD Ah^−1^. We further estimated the thickness of the DTH layer by calculating the net mass of deposited DTH molecules (∼ 2.9 mg, determined from residual ethanol solution after immersion treatment), yielding a maximum thickness of approximately 14 nm on the zinc plate. X‐ray photoelectron spectroscopy (XPS) measurements further verified the trace DTH adsorbate on the zinc surface. The N 1*s* and S 2*p* core‐level signals, initially enhanced with extended treatment, were no longer detectable after 30 s of electron beam etching (Figure S19), corroborating the above conclusion.

### 2.4 Full Cells

**FIGURE 5:**
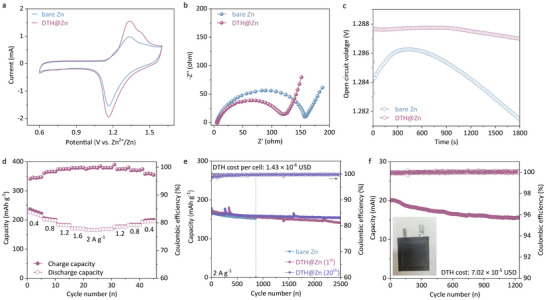
Electrochemical performance of Zn deposition in full cells. **a** CV curves (recorded at 5 mV s^−1^), **b** EIS spectra, **c** the plot of open circuit voltage as a function of time for Zn||ODASnI_4_ batteries which coupled with “bare Zn” or DTH@Zn; **d** rate performance of DTH@Zn||ODASnI_4_; **e** long‐term cycling performance of the coin cell assembled with the first and 20^th^ DTH treated Zn anode, and **f** the associated pouch cell. Inset in panel **f** is the photograph of the pouch cell.

To investigate the influence of the DTH layer on the cycling durability of full cells, Zn||ODASnI_4_ (ODA denotes 1,8‐octanediaminium cations) full batteries were assembled [58, 59, 60]. CV spectra recorded at 5 mV s^−1^ in Figure 5a emphasize the reversible conversion from I^0^/I^−^ regardless of the type of zinc anode. However, the use of DTH@Zn featured the cell with lower charge transfer resistance primarily due to the hindered corrosion of zinc and limited formation of insulating byproducts (Figure 5b). Besides, the nanoscale DTH layer exhibited a reasonable ability to exclude the contact between polyiodide ions and reductive zinc metal, thereby mitigating the shuttle effect and capacity loss to some extent and ensuring steady open circuit voltage for 1800 s, as shown in Figure 5c. The presence of DTH on the surface of the cycled Zn anode, confirmed by N1s core level XPS spectra (Figure S20), demonstrates robust adsorption. Electrostatic forces likely enhance this adsorption, facilitating the interaction of hydrophobic DTH with the zinc surface in the aqueous electrolyte. The attached DTH also contributed significantly to the rate performance (Figure 5d and Figure S21). In particular, the DTH@Zn||ODASnI_4_ battery delivered a high capacity of 227 mAh g^−1^_I_ at 0.4 A g^−1^ and 165 mAh g^−1^_I_ at 2 A g^−1^, which rose to 195 mAh g^−1^_I_ following the current reset, indicating a capacity retention of 85.9%. The long‐term performance of DTH@Zn||ODASnI_4_ was assessed with a focus on the first and 20^th^ samples from one batch of 5‐s DTH treatment, corresponding to an additive cost of 1.43× 10^−6^ USD per cell. As shown in Figure 5e, they both sustained 2500 cycles at the current density of 2 A g^−1^ with an average CE of 99.6%, meanwhile retaining capacity of 154 mAh g^−1^_I_ and 142 mAh g^−1^_I_ for batteries coupled with the first and 20^th^ DTH@Zn, respectively. The durable cycling performance and high capacity retention confirm the effectiveness of the DTH coating in suppressing parasitic reactions near the zinc anode while maintaining cathode electrode performance. Conversely, the control sample labeled “bare Zn” experienced a short circuit and failed after 800 cycles. Moreover, featuring the optimized configuration and a low DTH cost (7.02× 10^−5^ USD), the rigid pouch cell exhibited 1200 cycles at 3.3 mA cm^−2^, delivering a capacity of 19.95 mAh and a high energy density of 143 Wh kg^−1^ (based on the cathode material mass), with an average CE of 99.9% and 72% of its initial capacity retained (Figure 5f). The compatibility of the anode treatment strategy was further demonstrated by a flexible pouch cell employing the same electrode configuration, which successfully powered a cold‐light panel, as shown in Figure S22. The findings underscored the benefits of a hydrophobic DTH layer, emphasizing both its cost‐effectiveness and its critical role in stabilizing Zn chemistry.

## 3 Conclusion

We developed a facile surface treatment strategy to deposit a nanoscale DTH layer on the zinc surface, with a view to tackling undesired side reactions such as the growth of dendrites and HER. Being water‐resistant, a small amount of DTH was dissolved in an EtOH solution, after which the Zn electrode was immersed in this solution for several seconds to enable successive nanoscale coating of DTH for 20 zinc plates with a thickness below 14 nm, which corresponds to DTH dosage of 5.37 × 10^−13^ M and an ultra‐low CPC of 1.43 × 10^−7^ USD Ah^−1^, outperforming reported counterparts regarding both feeding dosage and additive costs. As a result, the assembled DTH@Zn||Cu asymmetric cell realized a life span of 2400 h (1200 cycles) and 2000 h (10,000 cycles) at 1 mA cm^−2^, 1 mAh cm^−2^, and 10 mA cm^−2^, 1 mAh cm^−2^, respectively. The DTH@Zn||DTH@Zn symmetric cell could sustain 2000 h of operation at 10 mA cm^−2^, 4 mAh cm^−2^. Moreover, both the first and 20^th^ samples, derived from the same batch of DTH treatment, realized an ultra‐low additive cost of 1.43× 10^−6^ USD per cell and successful operation for over 2500 cycles at 2 A g^−1^ in the configuration of DTH@Zn||ODASnI_4_ full cell. In contrast, the control sample based on “bare Zn” experienced a sudden short‐circuit and battery failure after 800 cycles. The rigid pouch cell further showcased the configuration's cost‐effectiveness (7.02× 10^−5^ USD) and durability, delivering 1200 cycles with 72% capacity retention while achieving a high energy density of 143 Wh kg^−1^ (based on cathode material mass).

## 4 Experimental Details

### 4.1 Chemicals and Reagents

1,3‐Di(o‐tolyl)thiourea (DTH, 98%), Zn sulfate heptahydrate (99%), Ethanol (EtOH, 99.5%), 1,8‐octadiamine (ODA, 98%), Tin iodide (SnI_2_, 99%) 1‐Methyl‐2‐pyrrolidinone (NMP, 98%), acrylamide (99%), potassium persulfate (99%), and N, N'‐methylenebisacrylamide (99%) were purchased from Aladdin. Polyvinylidene difluoride (PVDF) and Ketjen black EC‐300J were purchased from SOLVAY (Solef 1008) and Nouryon, respectively. All chemicals were used as received without further treatment.

### 4.2 Cell Fabrication

10 mg DTH was first dissolved in 20 mL EtOH solution and experienced ultrasonic treatment for an hour, followed by centrifugation at 2000 rpm for 15 min. The supernatant was retained for immersion treatment, during which zinc electrodes were submerged in the solution for specific durations (1 s, 5 s, or 5 min) and subsequently rinsed with distilled water. ODASnI_4_ was prepared using the saturated recrystallization method. The obtained perovskite microcrystals were mixed with Ketjen black and PVDF with a mass ratio of 7:2:1, followed by a grinding treatment with NMP for 1 h and dried under vacuum at 80 °C. The Zn||Zn symmetric coin cells were assembled by two (DTH‐treated) Zn foils (100 µm) and an intermediate glass fiber separator (Whatman, GF/C) with the addition of the 2 M ZnSO_4_ electrolyte (BZS). The Zn||Cu asymmetric cells, Zn||ODASnI_4_ coin and pouch cells followed a similar method. The pouch cell (4 × 6 cm^2^) had an active material loading of 6.25 mg cm^−2^, and the cell was cycled at a current density of 3.3 mA cm^−2^. The hydrogel electrolyte used in the flexible pouch battery was fabricated as follows: 5 g acrylamide, 15 mg potassium persulfate, 3 mg N,N'‐methylenebisacrylamide, and 2 M ZnSO_4_ were dissolved in 30 mL deionized water. The solution was stirred for 1 h, poured into a glass mold, and heated at 75 °C for 1 h to complete polymerization.

### 4.3 Characterization

Fourier‐transform infrared (FTIR) measurement was conducted using a Perkin‐Elmer FT‐IR spectrophotometer. Raman measurement was recorded using WITec RAMAN alpha 300R. Powder X‐ray diffraction (XRD) patterns were analyzed using a Rigaku Smartlab X‐ray diffractometer with Cu Kα radiation (*λ* = 1.5406 Å). The contact angle measurement was performed using a DataPhysics contact angle tester. X‐ray photoelectron spectroscopy (XPS) was carried out by a PHI model 5802; the carbon spectrum (284.8 eV) was used as a reference for calibration. The spot size used for XPS measurement was 650 µm. The morphology and elemental composition of the samples were studied using an FEI Quanta 250 scanning electron microscope (SEM) and energy dispersive spectroscopy (EDS). Cyclic voltammograms (CVs) and electrochemical impedance spectroscopy (EIS) were recorded on an electrochemical workstation (CHI 660E). The long‐term stability and rate performance of batteries were evaluated using the LAND battery testing system at room temperature.

### 4.4 Computation Methodology

The Dmol3 mode within a numerical atom‐centered basis function framework was adopted for the first‐principles calculations based on the density functional theory (DFT). The electronic exchange‐correlation interactions were resolved by the Perdew‐Burke‐Ernzerhof (PBE) method. The generalized gradient approximation (GGA) method with PBE formulation was used for the structural optimization [61, 62]. DFT semi‐core pseudopotentials were chosen for the core treatment with relativistic effects, which replaced the core electrons with a single effective potential. The adsorption energy *E_ads_* was evaluated through the equation: *E_ads_* = *E_ensemble_*  −*E_absorbent_* −*E_adsorbate_* where the subscript denotes either the energy of the absorbent, adsorbate, or the whole system after adsorption [63, 64, 65].

## Author Contributions

S. X. W. and C. Z. designed the study. C.Z. supervised the experiments. S. X. W, Z. W., Y. W., S. N. W., D. L., H. H., C. L., Y. W., Z. W., S. Z, X. Z., Y. C. L. conducted structural, electrochemical, and spectroscopic characterizations and analyzed the data. S. X. W. performed the theoretical calculations. All authors discussed the results and commented on the manuscript.

## Conflicts of Interest

The authors declare no conflicts of interest.

## Supporting information



Supporting Information

## Data Availability

The data that support the findings of this study are available from the corresponding author upon reasonable request.

## References

[1] X. Ji , and L. F. Nazar , “Best Practices for Zinc Metal Batteries,” Nature Sustainability 7 (2024): 98–99, 10.1038/s41893-023-01257-8.

[2] S. W. D. Gourley , R. Brown , B. D. Adams , and D. Higgins , “Zinc‐Ion Batteries for Stationary Energy Storage,” Joule 7 (2023): 1415–1436.

[3] S. Jin , J. Yin , X. Gao , et al., “Production of Fast‐Charge Zn‐Based Aqueous Batteries via Interfacial Adsorption of Ion‐Oligomer Complexes,” Nature Communications 13 (2022): 2283, 10.1038/s41467-022-29954-6. PMC9046403 35477721

[4] Z. Wang , J. Huang , Z. Guo , et al., “A Metal‐Organic Framework Host for Highly Reversible Dendrite‐free Zinc Metal Anodes,” Joule 3 (2019): 1289–1300, 10.1016/j.joule.2019.02.012.

[5] M. J. Counihan , K. S. Chavan , P. Barai , et al., “The Phantom Menace of Dynamic Soft‐Shorts in Solid‐State Battery Research,” Joule 8 (2024): 64–90, 10.1016/j.joule.2023.11.007.

[6] Y. Yang , H. Yang , R. Zhu , and H. Zhou , “High Reversibility at High Current Density: The Zinc Electrodeposition Principle Behind the 'Trick”, Energy & Environmental Science 16 (2023): 2723–2731, 10.1039/D3EE00925D.

[7] J. Luo , L. Xu , Y. Yang , et al., “Stable Zinc Anode Solid Electrolyte Interphase via Inner Helmholtz Plane Engineering,” Nature Communications 15 (2024): 6471, 10.1038/s41467-024-50890-0. PMC11291733 39085235

[8] M. Zhu , et al., “A Patternable and in Situ Formed Polymeric Zinc Blanket for a Reversible Zinc Anode in a Skin‐Mountable Microbattery,” Advanced Materials 33 (2021): e2007497. 33448064 10.1002/adma.202007497 PMC11469133

[9] Z. Pei , “Symmetric Is Nonidentical: Operation History Matters for Zn Metal Anode,” Nano Research Energy 1 (2022): e9120023, 10.26599/NRE.2022.9120023.

[10] Y. Kim , Y. Park , M. Kim , J. Lee , K.i J. Kim , and J. W. Choi , “Corrosion as the Origin of Limited Lifetime of Vanadium Oxide‐Based Aqueous Zinc Ion Batteries,” Nature Communications 13 (2022): 2371, 10.1038/s41467-022-29987-x. PMC9061739 35501314

[11] Y. Zhao , S. Guo , M. Chen , et al., “Tailoring Grain Boundary Stability of Zinc‐Titanium Alloy for Long‐Lasting Aqueous Zinc Batteries,” Nature Communications 14 (2023): 7080, 10.1038/s41467-023-42919-7. PMC10625522 37925505

[12] H. Peng , et al., “Surface Engineering on Zinc Anode for Aqueous Zinc Metal Batteries,” Chemsuschem 17 (2024): e202400076. 38429246 10.1002/cssc.202400076

[13] S. Liu , M. Maisuradze , M. Li , Q. Li , N. Kazemi , and M. Giorgetti , “Zincophilic MOF Protective Layer for Stable Zinc Anodes in Zinc‐Ion Batteries,” Chemistry European Journal 31 (2025): e02217, 10.1002/chem.202502217. PMC12520058 40847525

[14] C. Yang , P. Woottapanit , S. Geng , et al., “Biomimetic Inorganic–Organic Protective Layer for Highly Stable and Reversible Zn Anodes,” ACS Energy Letters 10 (2025): 337–344, 10.1021/acsenergylett.4c03005.

[15] T.‐B. Song , Q.‐L.i Ma , X.i‐R. Zhang , J.‐W. Ni , T.‐L.e He , and H.‐M. Xiong , “Zn Anode Surface Engineering for Stable Zinc‐Ion Batteries: Carbon Dots Incorporated Mesoporous TiO_2_ as a Coating Layer,” Chemistry Engineering Journal 471 (2023): 144735, 10.1016/j.cej.2023.144735.

[16] Y.u Zong , H. He , Y. Wang , et al., “Functionalized Separator Strategies Toward Advanced Aqueous Zinc‐Ion Batteries,” Advanced Energy Materials 13 (2023): 2300403, 10.1002/aenm.202300403.

[17] S. Bhoyate , S. Mhin , J.‐E. Jeon , K. Park , J. Kim , and W. Choi , “Stable and High‐Energy‐Density Zn‐Ion Rechargeable Batteries Based on a MoS_2_‐Coated Zn Anode,” ACS Applies Materials Interfaces 12 (2020): 27249–27257, 10.1021/acsami.0c06009. 32437120

[18] Y. Geng , L. Pan , Z. Peng , et al., “Electrolyte Additive Engineering for Aqueous Zn Ion Batteries,” Energy Storage Materials 51 (2022): 733–755, 10.1016/j.ensm.2022.07.017.

[19] J. Yin , Y. Wang , Y. Zhu , et al., “Regulating the Redox Reversibility of Zinc Anode Toward Stable Aqueous Zinc Batteries,” Nano Energy 99 (2022): 107331, 10.1016/j.nanoen.2022.107331.

[20] J. Yang , B. Yin , Y. Sun , et al., “Zinc Anode for Mild Aqueous Zinc‐Ion Batteries: Challenges, Strategies, and Perspectives,” Nanomicro Letters 14 (2022): 42. 10.1007/s40820-021-00782-5 PMC8724388 34981202

[21] R. Qin , Y. Wang , L. Yao , et al., “Progress in Interface Structure and Modification of Zinc Anode for Aqueous Batteries,” Nano Energy 98 (2022): 107333.

[22] Z. Wu , J. Zou , Y. Li , et al., “Regulating Zinc Nucleation Sites and Electric Field Distribution to Achieve High‐Performance Zinc Metal Anode via Surface Texturing,” Small 19 (2022): e2206634. 36437113 10.1002/smll.202206634

[23] N. Guo , W. Huo , X. Dong , et al., “A Review on 3D Zinc Anodes for Zinc Ion Batteries,” Small Methods 6 (2022): e2200597, 10.1002/smtd.202200597. 35853247

[24] R. Wang , M. Yao , M. Yang , J. Zhu , J. Chen , and Z. Niu , “Synergetic Modulation on Ionic Association and Solvation Structure by Electron‐Withdrawing Effect for Aqueous Zinc‐Ion Batteries,” Proceedings National Academy of Science USA 120 (2023): e2221980120, 10.1073/pnas.2221980120. PMC10104530 37023128

[25] L. Cao , D. Li , E. Hu , et al., “Solvation Structure Design for Aqueous Zn Metal Batteries,” Journal of the American Chemical Society 142 (2020): 21404–21409, 10.1021/jacs.0c09794. 33290658

[26] F. Wu , Y. Chen , Y. Chen , et al., “Achieving Highly Reversible Zinc Anodes via N, N‐Dimethylacetamide Enabled Zn‐Ion Solvation Regulation,” Small 18 (2022): e2202363, 10.1002/smll.202202363. 35665600

[27] J. Yang , Y. Zhang , Z. Li , et al., “Three Birds With One Stone: Tetramethylurea as Electrolyte Additive for Highly Reversible Zn‐Metal Anode,” Advanced Functional Materials 32 (2022): 2209642, 10.1002/adfm.202209642.

[28] H. Ge , X. Feng , D. Liu , and Y. Zhang , “Recent Advances and Perspectives for Zn‐Based Batteries: Zn Anode and Electrolyte,” Nano Res Energy 2 (2023): e9120039, 10.26599/NRE.2023.9120039.

[29] J. Abdulla , J. Cao , D. Zhang , et al., “Elimination of Zinc Dendrites by Graphene Oxide Electrolyte Additive for Zinc‐Ion Batteries,” ACS Applied Energy Materials 4 (2021): 4602–4609, 10.1021/acsaem.1c00224.

[30] H. Wang , H. Li , Y. Tang , et al., “Stabilizing Zn Anode Interface by Simultaneously Manipulating the Thermodynamics of Zn Nucleation and Overpotential of Hydrogen Evolution,” Advanced Functional Materials 32 (2022): 2207898, 10.1002/adfm.202207898.

[31] M. Qiu , P. Sun , G. Cui , and W. Mai , “Chaotropic Polymer Additive With Ion Transport Tunnel Enable Dendrite‐Free Zinc Battery,” ACS Applied Materials Interfaces 14 (2022): 40951–40958, 10.1021/acsami.2c10517. 36039409

[32] I. D. Brown , “Structural Chemistry and Solvent Properties of Dimethylsulfoxide,” Journal of Solution Chemistry 16 (1987): 205–224, 10.1007/BF00646987.

[33] D. Yao , Yi Liu , W. Zhao , et al., “A Totally Phosphine‐Free Synthesis of Metal Telluride Nanocrystals by Employing Alkylamides to Replace Alkylphosphines for Preparing Highly Reactive Tellurium Precursors,” Nanoscale 5 (2013): 9593, 10.1039/c3nr03553k. 24056800

[34] M. M. Davis , Acid‐Base Behavior in Aprotic Organic Solvents. (United States. Government Printing Office, 1968).

[35] H. Jin , S. Dai , Z. Zhu , et al., “Crystal Water Boosted Zn^2+^ Transfer Kinetics in Artificial Solid Electrolyte Interphase for High‐Rate and Durable Zn Anodes,” ACS Applied Energy Materials 5 (2022): 10581–10590, 10.1021/acsaem.2c01340.

[36] D. Xie , Y. Sang , D.‐H. Wang , et al., “ZnF_2_ ‐Riched Inorganic/Organic Hybrid SEI: In Situ‐Chemical Construction and Performance‐Improving Mechanism for Aqueous Zinc‐ion Batteries,” Angewandte Chemie International Edition 62 (2023): e202216934, 10.1002/anie.202216934. 36478517

[37] G. Liang , J. Zhu , B. Yan , et al., “Gradient Fluorinated Alloy to Enable Highly Reversible Zn‐Metal Anode Chemistry,” Energy & Environmental Science 15 (2022): 1086–1096, 10.1039/D1EE03749H.

[38] Y. Zhang , S. Shen , K. Xi , et al., “Suppressed Dissolution of Fluorine‐Rich SEI Enables Highly Reversible Zinc Metal Anode for Stable Aqueous Zinc‐Ion Batteries,” Angewandte Chemie International Edition 63 (2024): e202407067, 10.1002/anie.202407067. 38771481

[39] K. Wu , et al., “Regulating Zn Deposition via an Artificial Solid‐Electrolyte Interface With Aligned Dipoles for Long Life Zn Anode,” Nanomicro Letters 13 (2021): 79. 10.1007/s40820-021-00599-2 PMC8187518 34138325

[40] H. Lu , X. Zhang , M. Luo , et al., “Amino Acid‐Induced Interface Charge Engineering Enables Highly Reversible Zn Anode,” Advanced Functional Materials 31 (2021): 2103514, 10.1002/adfm.202103514.

[41] F. Yang , J. A. Yuwono , J. Hao , et al., “Understanding H 2 Evolution Electrochemistry to Minimize Solvated Water Impact on Zinc‐Anode Performance,” Advanced Materials 34 (2022): e2206754, 10.1002/adma.202206754. 36124561

[42] S. Wang , S. Wang , Z. Wei , et al., “A Parts‐Per‐Million Scale Electrolyte Additive for Durable Aqueous Zinc Batteries,” Nature Communications 16 (2025): 1800, 10.1038/s41467-025-56607-1. PMC11842810 39979314

[43] S. Wang , J. Popovic , S. Burazer , et al., “Strongly Luminescent Dion–Jacobson Tin Bromide Perovskite Microcrystals Induced by Molecular Proton Donors Chloroform and Dichloromethane,” Advanced Functional Materials 31 (2021): 2102182, 10.1002/adfm.202102182.

[44] S. Wang , Z. Huang , J. Zhu , et al., “Quantifying Asymmetric Zinc Deposition: A Guide Factor for Designing Durable Zinc Anodes,” Advanced Materials 36 (2024): e2406451, 10.1002/adma.202406451. 38888505

[45] H.u Hong , J. Zhu , Y. Wang , et al., “Metal‐Free Eutectic Electrolyte With Weak Hydrogen Bonds for High‐Rate and Ultra‐Stable Ammonium‐Ion Batteries,” Advanced Materials 36 (2024): e2308210, 10.1002/adma.202308210. 37916840

[46] T. H. Wan , M. Saccoccio , C. Chen , and F. Ciucci , “Influence of the Discretization Methods on the Distribution of Relaxation Times Deconvolution: Implementing Radial Basis Functions With DRTtools,” Electrochimica Acta 184 (2015): 483–499, 10.1016/j.electacta.2015.09.097.

[47] R. Soni , J. B. Robinson , P. R. Shearing , D. J. L. Brett , A. J. E. Rettie , and T. S. Miller , “Lithium‐Sulfur Battery Diagnostics Through Distribution of Relaxation Times Analysis,” Energy Storage Materials 51 (2022): 97–107, 10.1016/j.ensm.2022.06.016.

[48] L. Ma , M. A. Schroeder , O. Borodin , et al., “Realizing High Zinc Reversibility in Rechargeable Batteries,” Nature Energy 5 (2020): 743–749, 10.1038/s41560-020-0674-x.

[49] A. Bayaguud , X. Luo , Y. Fu , and C. Zhu , “Cationic Surfactant‐Type Electrolyte Additive Enables Three‐Dimensional Dendrite‐Free Zinc Anode for Stable Zinc‐Ion Batteries,” ACS Energy Letters 5 (2020): 3012–3020, 10.1021/acsenergylett.0c01792.

[50] X. Guo , Z. Zhang , J. Li , et al., “Alleviation of Dendrite Formation on Zinc Anodes via Electrolyte Additives,” ACS Energy Letters 6 (2021): 395–403, 10.1021/acsenergylett.0c02371.

[51] M. Shi , C. Lei , H. Wang , et al., “Molecule Engineering of Sugar Derivatives as Electrolyte Additives for Deep‐Reversible Zn Metal Anode,” Angewandte Chemie International Edition 63 (2024): e202407261, 10.1002/anie.202407261. 38842470

[52] M. Li , K. Xie , R. Peng , B. Yuan , Q. Wang , and C. Wang , “Surface Protection and Interface Regulation for Zn Anode via 1‐Hydroxy Ethylidene‐1,1‐Diphosphonic Acid Electrolyte Additive Toward High‐Performance Aqueous Batteries,” Small 18 (2022): e2107398, 10.1002/smll.202107398. 35083869

[53] H. Huang , D. Xie , J. Zhao , et al., “Boosting Reversibility and Stability of Zn Anodes via Manipulation of Electrolyte Structure and Interface With Addition of Trace Organic Molecules,” Advanced Energy Materials 12 (2022): 2202419, 10.1002/aenm.202202419.

[54] Q.i Meng , Q. Bai , R. Zhao , et al., “Attenuating Water Activity through Impeded Proton Transfer Resulting From Hydrogen Bond Enhancement Effect for Fast and Ultra‐Stable Zn Metal Anode,” Advanced Energy Materials 13 (2023): 2302828, 10.1002/aenm.202302828.

[55] T. Wei , Y. Ren , Y. Wang , et al., “Addition of Dioxane in Electrolyte Promotes (002)‐Textured Zinc Growth and Suppressed Side Reactions in Zinc‐Ion Batteries,” ACS Nano 17 (2023): 3765–3775, 10.1021/acsnano.2c11516. 36752806

[56] K. Zhao , G. Fan , J. Liu , et al., “Boosting the Kinetics and Stability of Zn Anodes in Aqueous Electrolytes With Supramolecular Cyclodextrin Additives,” Journal of the American Chemical Society 144 (2022): 11129–11137, 10.1021/jacs.2c00551. 35700394

[57] D.‐S. Liu , Z. Zhang , Y. Zhang , et al., “Manipulating OH—‐Mediated Anode‐Cathode Cross‐Communication toward Long‐Life Aqueous Zinc‐Vanadium Batteries,” Angewandte Chemie International Edition 62 (2023): e202215385, 10.1002/anie.202215385. 36437231

[58] X. Li , S. Wang , D. Zhang , et al., “Perovskite Cathodes for Aqueous and Organic Iodine Batteries Operating Under One and Two Electrons Redox Modes,” Advanced Materials 36 (2024): e2304557, 10.1002/adma.202304557. 37587645

[59] S. Wang , Z. Huang , B. Tang , et al., “Conversion‐Type Organic‐Inorganic Tin‐Based Perovskite Cathodes for Durable Aqueous Zinc‐Iodine Batteries,” Advanced Energy Materials 13 (2023): 2300922, 10.1002/aenm.202300922.

[60] S. Wang , Y. Wang , Z. Wei , et al., “Halide Exchange in Perovskites Enables Bromine/Iodine Hybrid Cathodes for Highly Durable Zinc Ion Batteries,” Advanced Materials 36 (2024): e2401924, 10.1002/adma.202401924. 38593988

[61] W. Setyawan , S. Curtarolo , “High‐Throughput Electronic Band Structure Calculations: Challenges and Tools,” Computational Materials Science 49 (2010): 299–312.

[62] H. Zhu , R. J. Kee , “Computational Modeling of Sodium‐Iodine Secondary Batteries,” Electrochimica Acta 219 (2016): 70–81, 10.1016/j.electacta.2016.09.104.

[63] T. Lu , F. Chen , “Multiwfn: A Multifunctional Wavefunction Analyzer,” Journal of Computational Chemistry 33 (2012): 580–592, 10.1002/jcc.22885. 22162017

[64] S. Wang , R. Shi , B. Tang , et al., “Co‐Doping of Tellurium With Bismuth Enhances Stability and Photoluminescence Quantum Yield of Cs_2_AgInCl_6_ Double Perovskite Nanocrystals,” Nanoscale 14 (2022): 15691–15700, 10.1039/D2NR04717A. 36263792

[65] X. Li , S. Wang , T. Wang , et al., “Bis‐Ammonium Salts With Strong Chemisorption to Halide Ions for Fast and Durable Aqueous Redox Zn Ion Batteries,” Nano Energy 98 (2022): 107278, 10.1016/j.nanoen.2022.107278.

