# Supplementary material for: Ultra‐Low‐Cost Hydrophobic Organic Coating for Highly Reversible Zinc Anodes
Source: Angew Chem Int Ed Engl. 2026 Feb 3;65(11):e23567. doi: 10.1002/anie.202523567 (PMC12970510; doi:10.1002/anie.202523567)
Supplement: Supplementary file 1 — Supporting Information [file ANIE-65-e23567-s001.docx]

**Supplementary information**

**Ultra-low-cost hydrophobic organic coating for highly reversible zinc anodes**

Shixun Wang,^1,2^ Zhiquan Wei,^1^ Yiqiao Wang,^1^ Shengnan Wang,^1,2^ Dedi Li,^1^ Hu Hong,^1,2^ Chuan Li,^1,2^ Yanbo Wang,^1,2^ Zhuoxi Wu,^1,2^ Shaoce Zhang,^1,2^ Xueying Zheng,^1,2^ Yi-Chun Lu,^3*^ Chunyi Zhi^1,2^*

1 Department of Mechanical Engineering, The University of Hong Kong, Hong Kong SAR, China

2 Department of Materials Science and Engineering, City University of Hong Kong, Hong Kong S.A.R., 999077, P. R. China

3 Electrochemical Energy and Interfaces Laboratory, Department of Mechanical and Automation Engineering, The Chinese University of Hong Kong, Hong Kong SAR, China

* Corresponding authors: [yichunlu@mae.cuhk.edu.hk](mailto:yichunlu@mae.cuhk.edu.hk); cyzhi@hku.hk

**Supplementary Table 1**. Cost table of DTH and other additives reported in the literature.

| Types of additives | Price (USD g^-1^) | Additive amount (mol L^-1^, or M) | Cost of CPC  (USD Ah^-1^) | Refs |
| --- | --- | --- | --- | --- |
| GO | 27.340 | 1.27 × 10^-5^ | 0.1583 | 1 |
| HA | 2392.250 | 3.20 × 10^-5^ | 0.0695 | 2 |
| TBA^+^ | 0.860 | 5.00 × 10^-5^ | 0.0187 | 3 |
| LiCl | 0.220 | 2.00 | 0.0321 | 4 |
| DX | 1.640 | 6.26 × 10^-2^ | 0.0115 | 5 |
| GBL | 0.116 | 1.30 × 10^-1^ | 0.0003 | 6 |
| ET | 1.500 | 2.00 × 10^-1^ | 0.0059 | 7 |
| ARG | 0.180 | 1.00 × 10^-1^ | 2.4318 | 8 |
| Sericin | 223.880 | 5.28 × 10^-7^ | 0.0009 | 9 |
| TMU | 0.240 | 2.50 × 10^-1^ | 0.5300 | 10 |
| Glycine | 0.075 | 3.00 | 0.0053 | 11 |
| ASO | 0.048 | 2.00 × 10^-1^ | 0.2329 | 12 |
| LA | 0.340 | 7.00 × 10^-2^ | 0.0054 | 13 |
| DMSO | 0.031 | 2.53 | 0.0125 | 14 |
| CDs | 0.870 | 1.00 × 10^-2^ | 0.0019 | 15 |
| HEDP | 0.170 | 1.00 × 10^-3^ | 0.0020 | 16 |
| DMA | 0.071 | 1.08 | 0.0154 | 17 |
| DTH | 0.780 | 5.73 × 10^-13^ | 1.43 × 10^-7^ | This work |

Abbreviation: GO, Graphene oxide; HA, Hyaluronic acid; TBA^+^, Tetrabutylammonium sulfate; LiCl, lithium chloride; DX, 1,3-Dioxane; GBL, γ-Butyrolactone; ET, Ectoine; ARG, arginine; TMU, Tetramethylurea; ASO, Al_2_(SO_4_)_3_; LA, Lactobionic acid; DMSO, dimethyl sulfoxide; CDs, cyclodextrins; HEDP, 1-hydroxy ethylidene-1,1-diphosphonic acid; DMA, N,N-Dimethylacetamide.

**Supplementary Note 1**.

Detailed calculations for DTH layer thickness and values in the above table are provided below.

1. DTH concentration in the ethanol solution was calculated as:

$C_{DTH}=\frac{10 mg}{20 mL}=\frac{10 mg}{20 mL\times0.789 g {mL}^{-1}}\times100\%=0.0634\%$ (unitless, assuming normalized to g/g)

1. Residual DTH amount on the zinc plate after the immersion amount:

Upon withdrawing the immersed zinc electrode (0.7 × 0.7 cm^2^) from the DTH/ethanol solution, approximately 2.9 mg of the liquid solution remains adhered to the electrode surface, thereby yielding a rough net DTH amount of:

$$m_{DTH}=C_{DTH}\times2.9 mg=0.000634\times2.9 mg=0.0018386 mg$$

1. Given an electrolyte addition of 80 µL for each cell, the corresponding additive amount was approximately estimated as below, for comparative analysis with literature values only:

$$V_{DTH}=\frac{m_{DTH}}{M_{DTH}\times80\mu L}=\frac{m_{DTH}}{256.37 g {mol}^{-1}\times80\mu L}= 5.37 \times{10}^{-13} M$$

1. Cost per cumulative plated capacity (CPC):

The CPC was 10 Ah (**Fig. 3**e). The associated cost could be calculated as:

$${Cost}_{CPC}=m_{DTH}\times0.780 USDg^{-1}/10 Ah=1.43\times{10}^{-7} USD {Ah}^{-1}$$

1. Maximum thickness of DTH layer on one side of the zinc plate ($M_{DTH}=256.37 g {mol}^{-1}$, $N_{A}=6.02\times{10}^{23}{mol}^{-1}$,$x\approx1.2 nm$, $y\approx0.55 nm$, $z\approx0.5 nm$, where $x$, $y$, $z$ represents the Length, width and height of DTH molecule, respectively) was estimated to be:

$$D=\frac{{0.5\times m}_{DTH}\times M_{DTH}\times N_{A}}{0.49}\times xyz=44 {nm}^{-2}\times xyz\approx14 nm$$

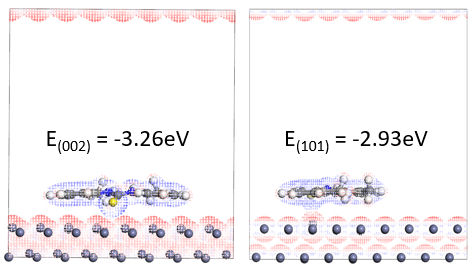


**Supplementary Fig. 1** Adsorption energy of DTH molecules at zinc surface.


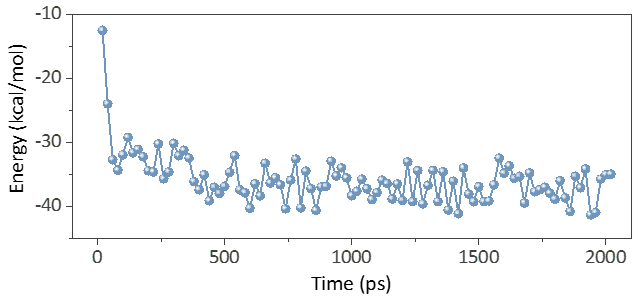


**Supplementary Fig. 2** The evolution of the total system energy of MD simulations in Fig. 2c.


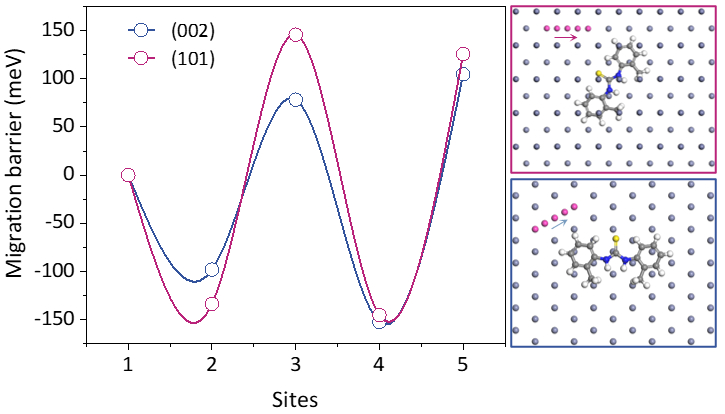


**Supplementary Fig. 3** Migration energy of zinc atom after the adsorption of DTH.


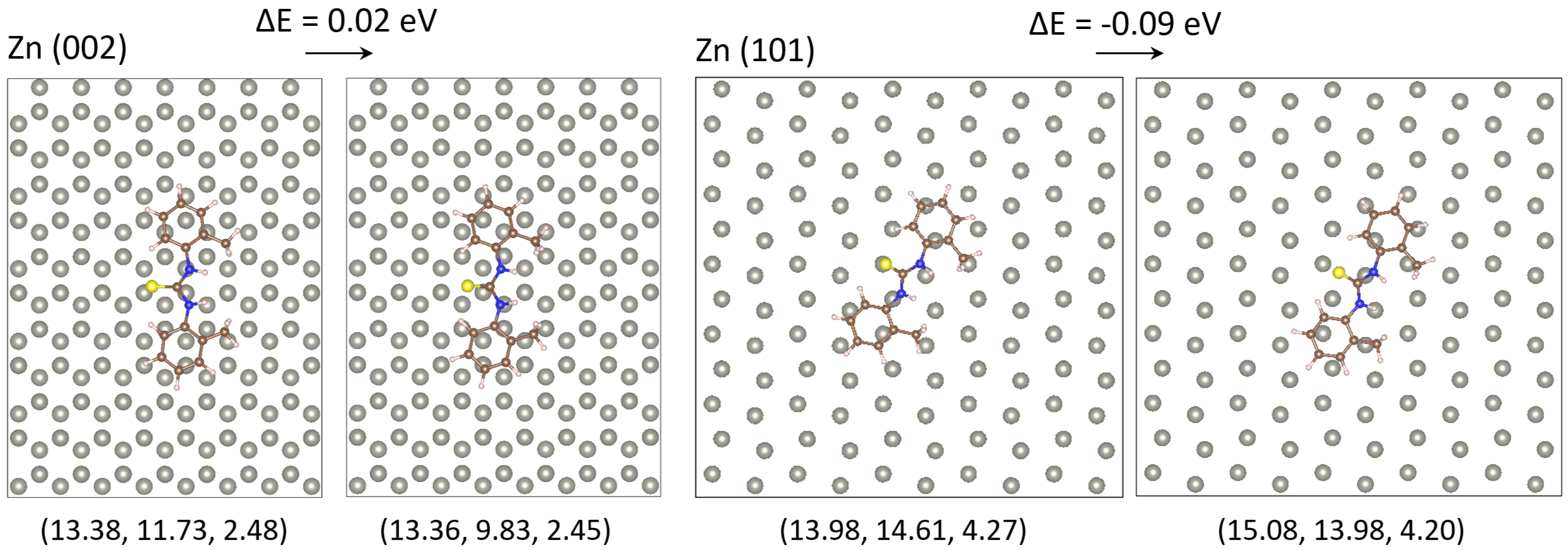


**Supplementary Fig. 4** Low migration energy of DTH on the surface of the (002) and (101) lattice planes of zinc metals. The geometrical center of DTH is demonstrated at the bottom of each frame.


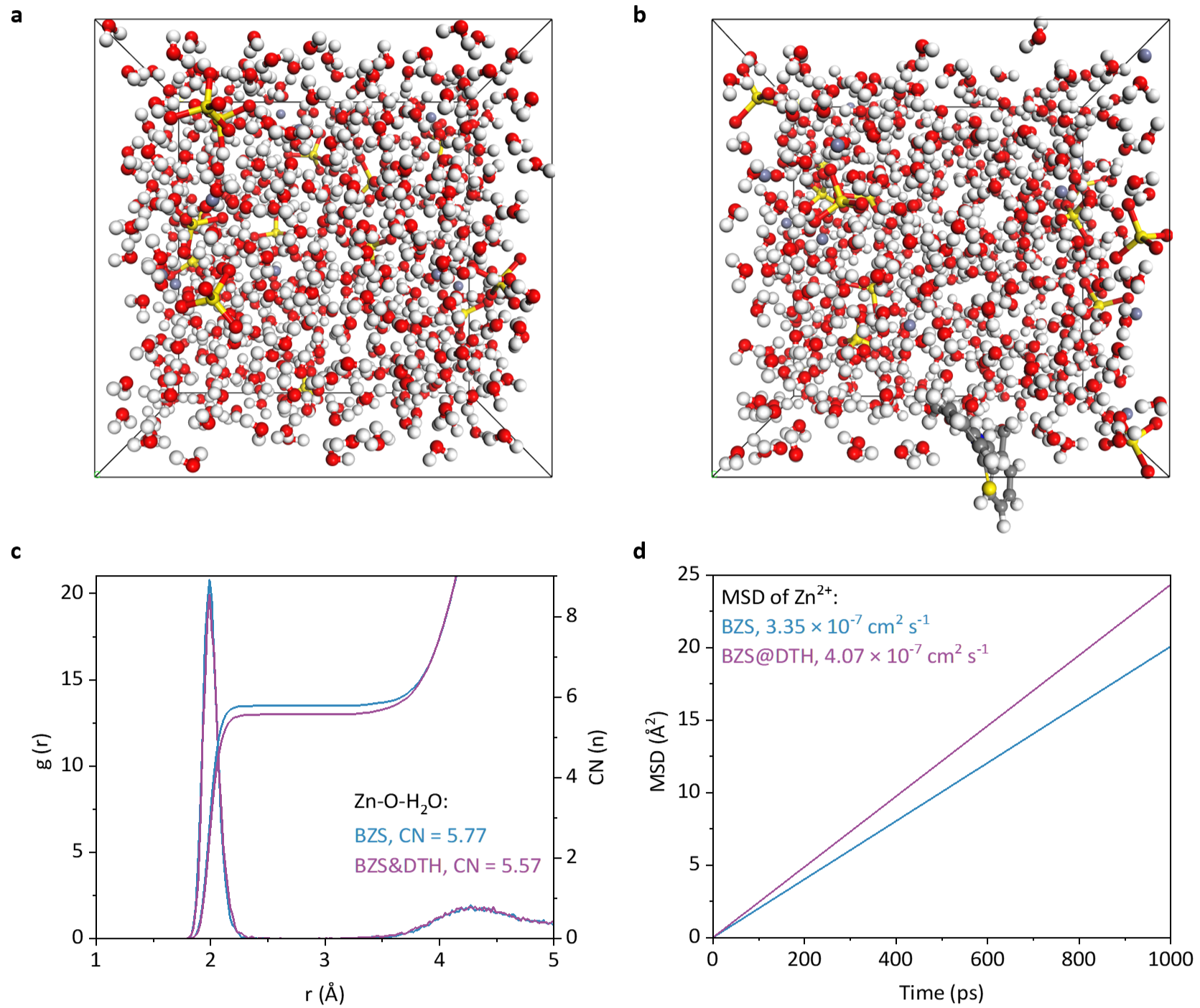


**Supplementary Fig. 5** 3D snapshot of **a** BZS and **b** 2.2 wt.% DTH in BZS from MD simulation (atoms in red, grey, white, pink and yellow represent oxygen, zinc, hydrogen, phosphorus and sulfur, respectively); **c** RDFs for Zn-O-H_2_O and **d** MSD of Zn^2+^ in the studied electrolytes.

Despite its hydrophobic nature, the inclusion of a single DTH molecule (2.2 wt.% relative to BZS) in a simplified electrolyte model should enable the study of Zn^2+^-water interactions near the DTH layer. We thus conducted molecular dynamics (MD) simulations to reveal the interfacial working mechanism of the DTH coating. For specific, the radial distribution function (RDF) analysis of the interaction between Zn^2+^ and O in H_2_O revealed that the average coordination number (CN) of Zn^2+^ decreased from 5.77 for BZS to 5.57 in the electrolyte containing DTH. This reduction indicates fewer coordinated water molecules in the first solvation shell of Zn^2+^, which promotes the desolvation process of Zn(H_2_O)_5.77_^2+^, particularly at the zinc surface where hydrophobic DTH molecules accumulate. MD simulations further demonstrated a slight decrease in the mean squared displacement (MSD) of Zn^2+^ upon DTH inclusion, corresponding to optimized Zn^2+^ mobility which could contribute to smoother zinc flux and more uniform deposition in the electrode/electrolyte interface.


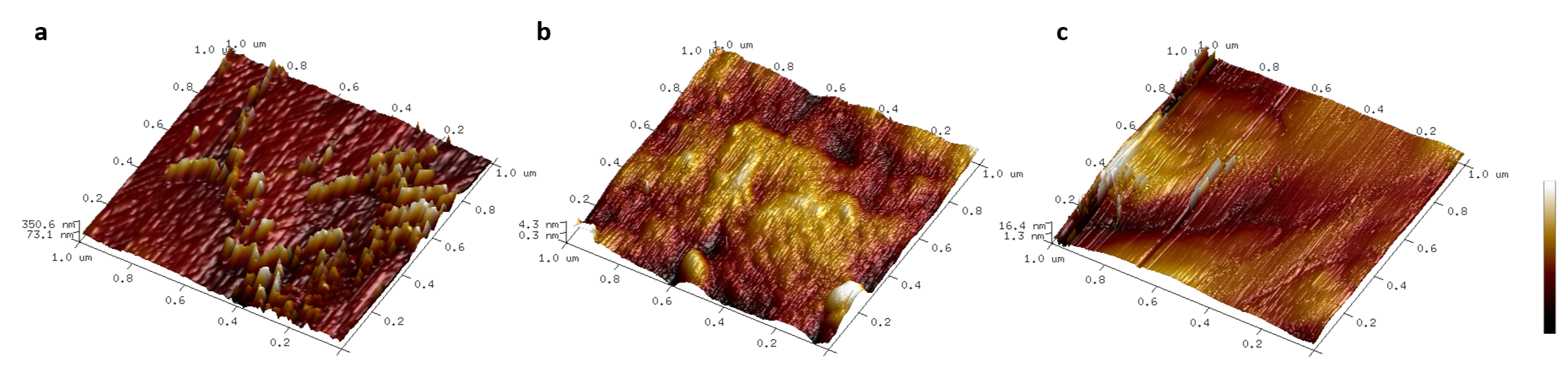


**Supplementary Fig. 6** Atomic force microscope images of **a** fresh Zn plate, **b** DTH@Zn, and **c** 5 min immersion treated Zn plate, in a region of 1 × 1 µm^2^.


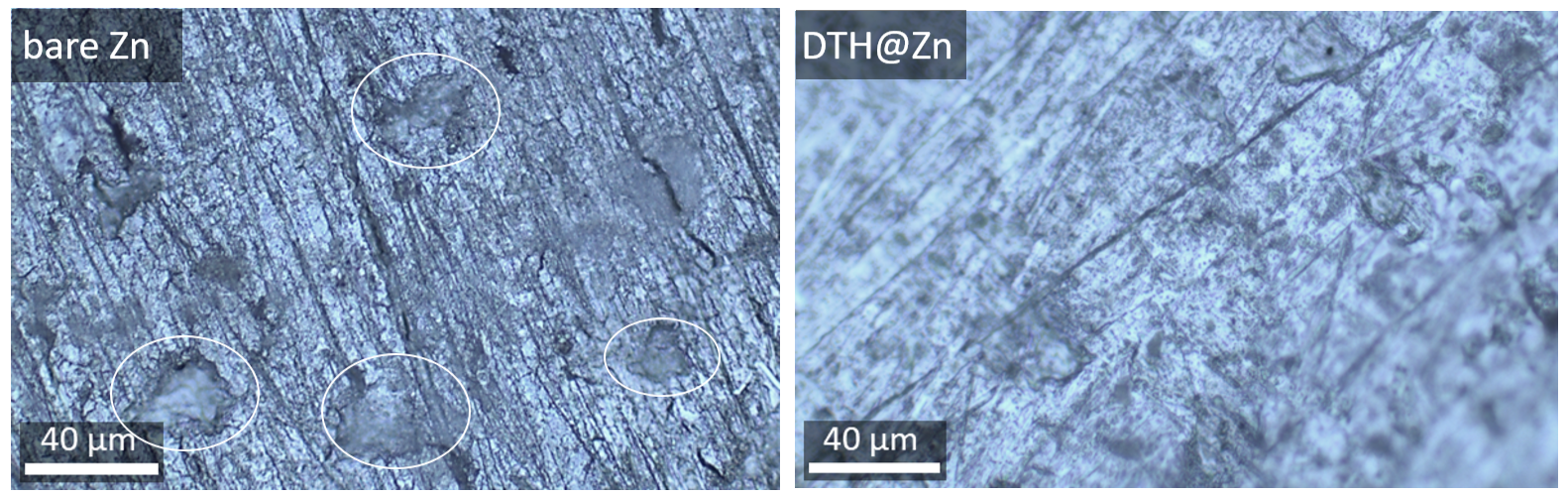


**Supplementary Fig. 7** Confocal optical microscopy images of bare Zn and DTH@Zn after the immersion treatment in 2 M ZnSO_4_ electrolyte.


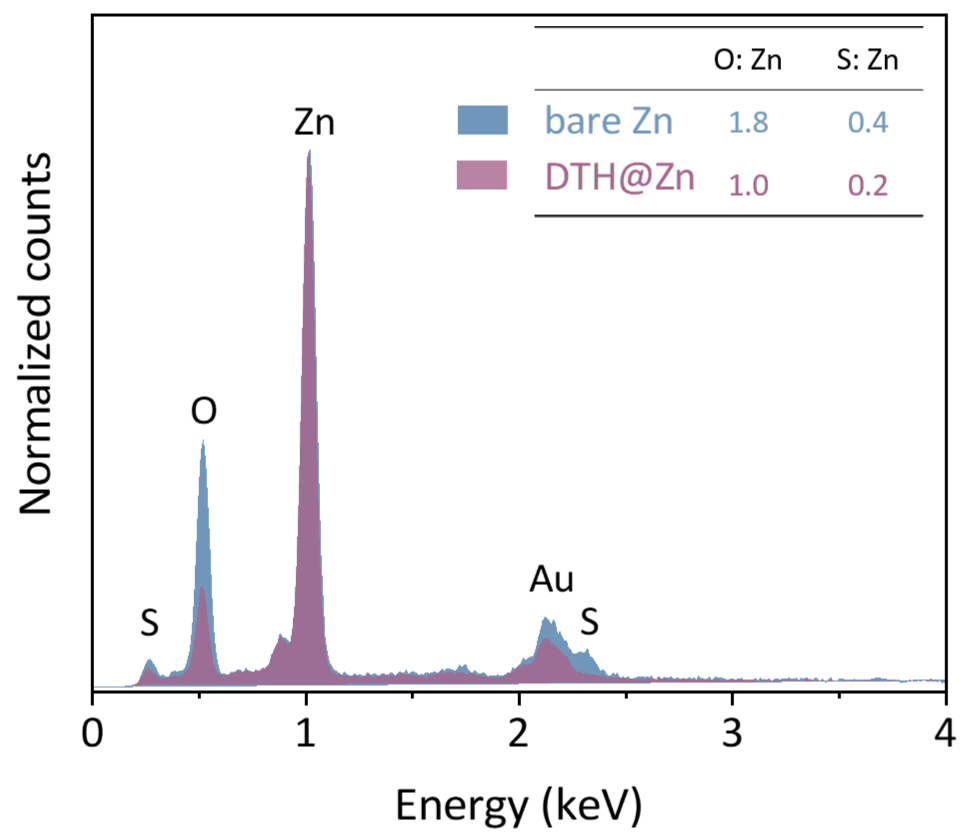


**Supplementary Fig. 8** EDS results of Bare Zn and DTH@Zn after the immersion treatment in 2 M ZnSO_4_ electrolyte.


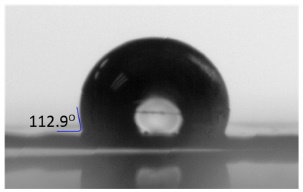


**Supplementary Fig. 9** Contact angle analysis of the Zn plate experienced 5 min DTH treatment.


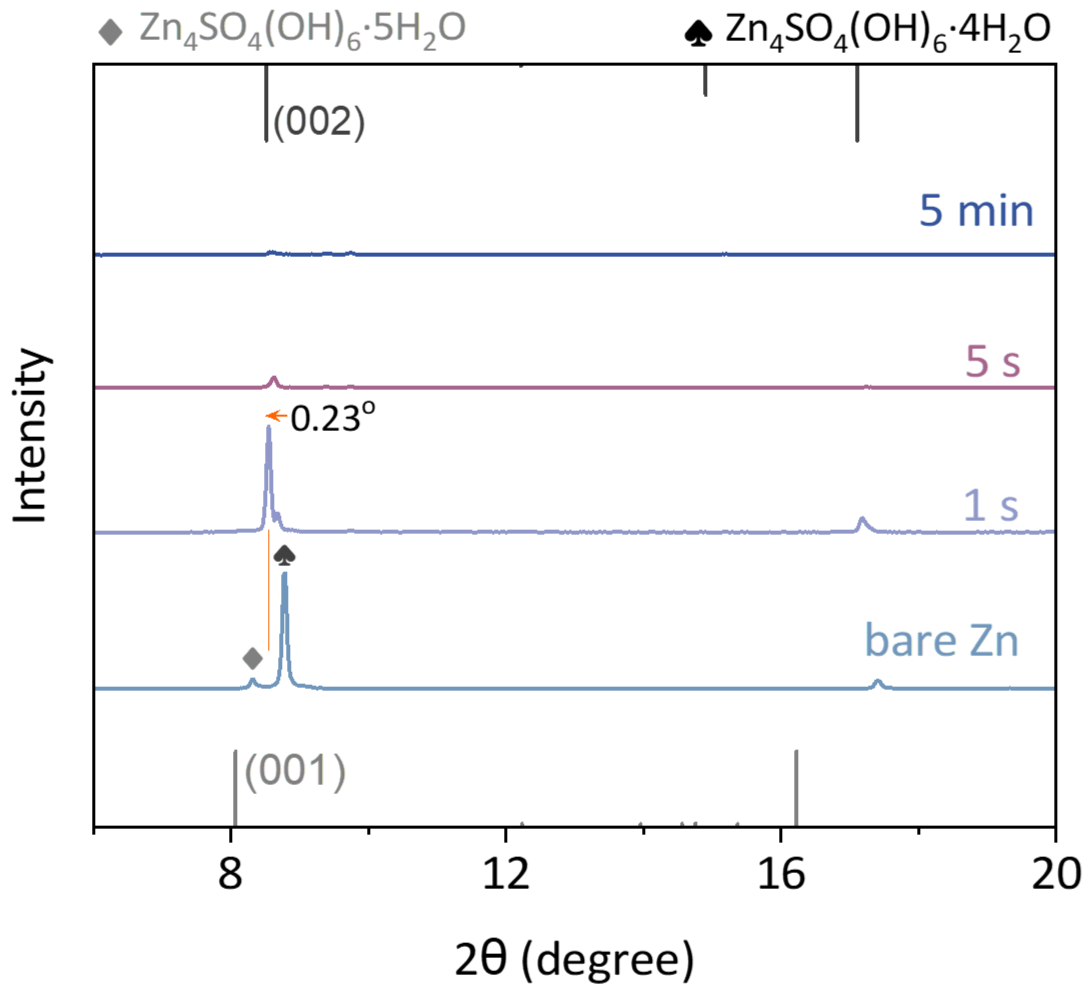


**Supplementary Fig. 10** Enlarged XRD pattern of Fig. 3e, which demonstrates "bare Zn" and DTH-treated Zn plates after immersion in BZS for 1 week.


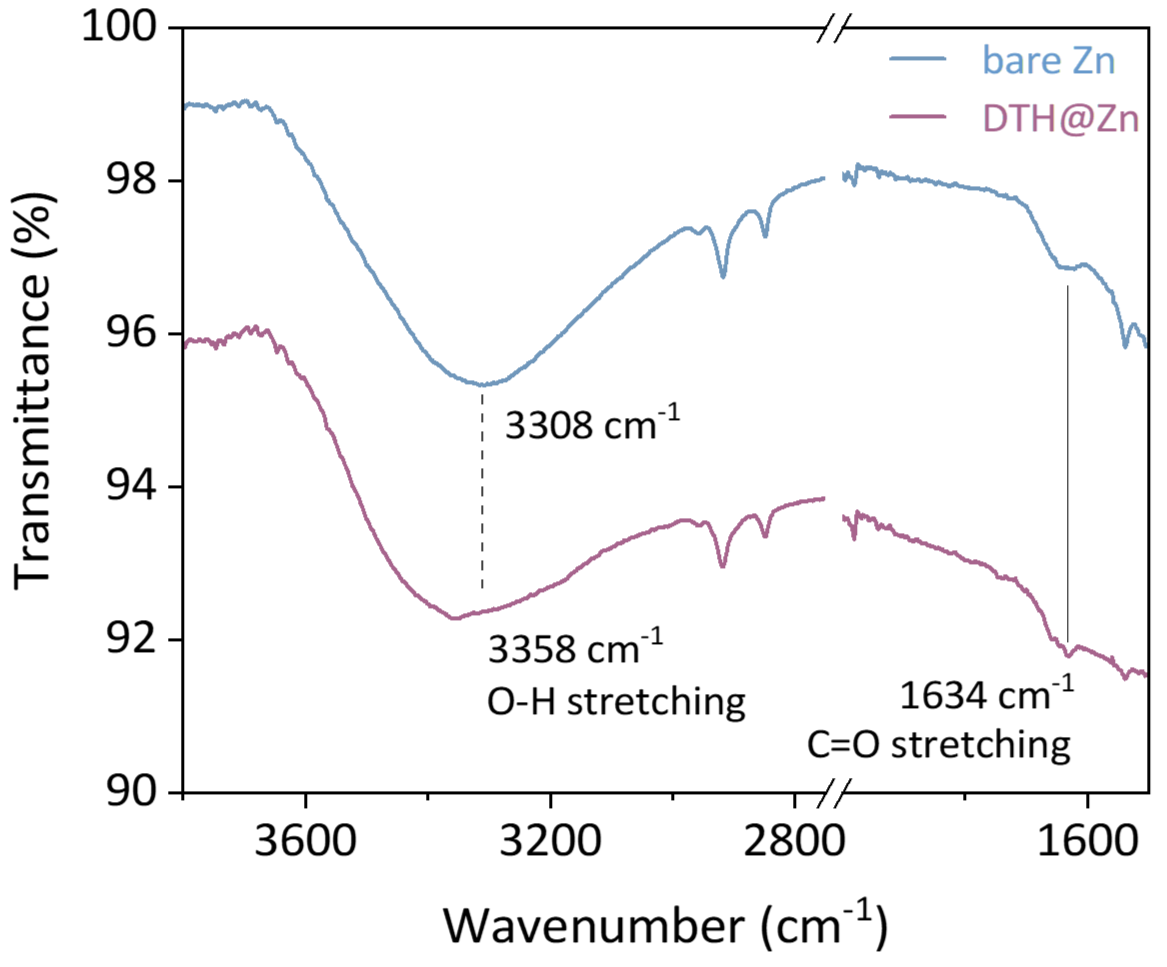


**Supplementary Fig. 11** FTIR spectra of bare Zn and DTH@Zn electrodes.


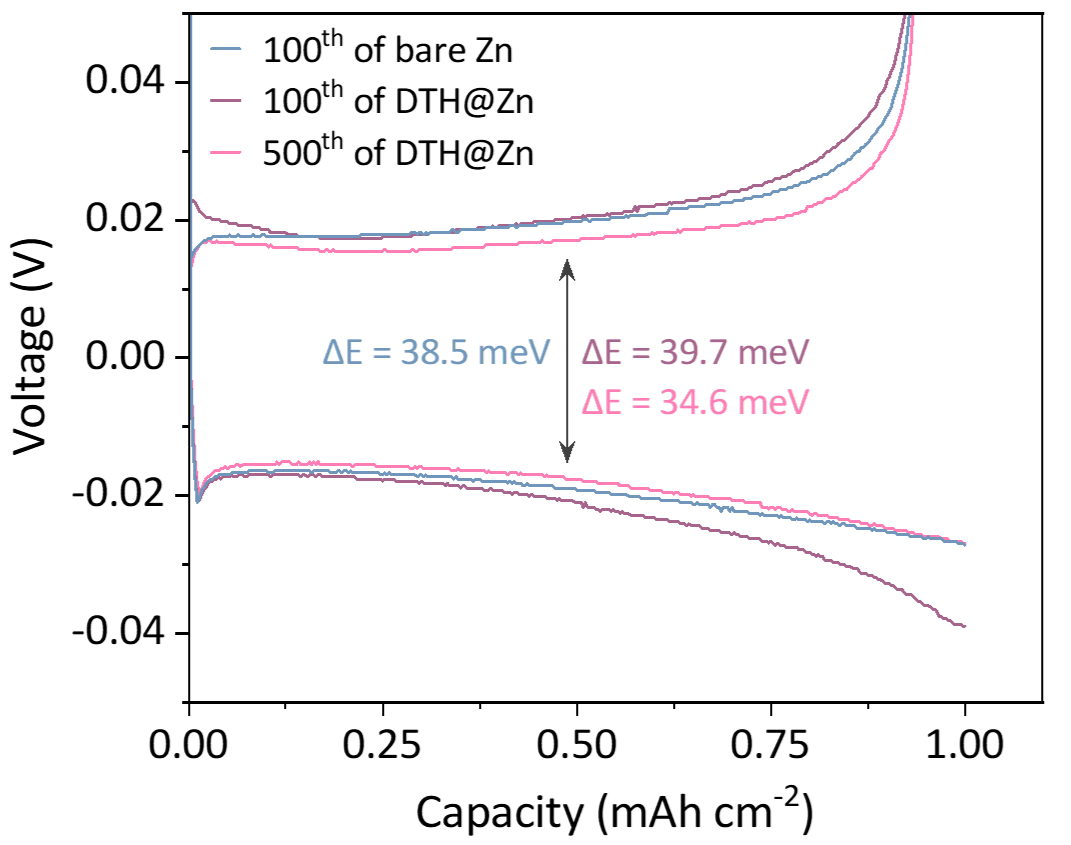


**Supplementary Fig. 12** The voltage profile of cycled bare Zn||Cu and DTH@Zn||Cu cells.


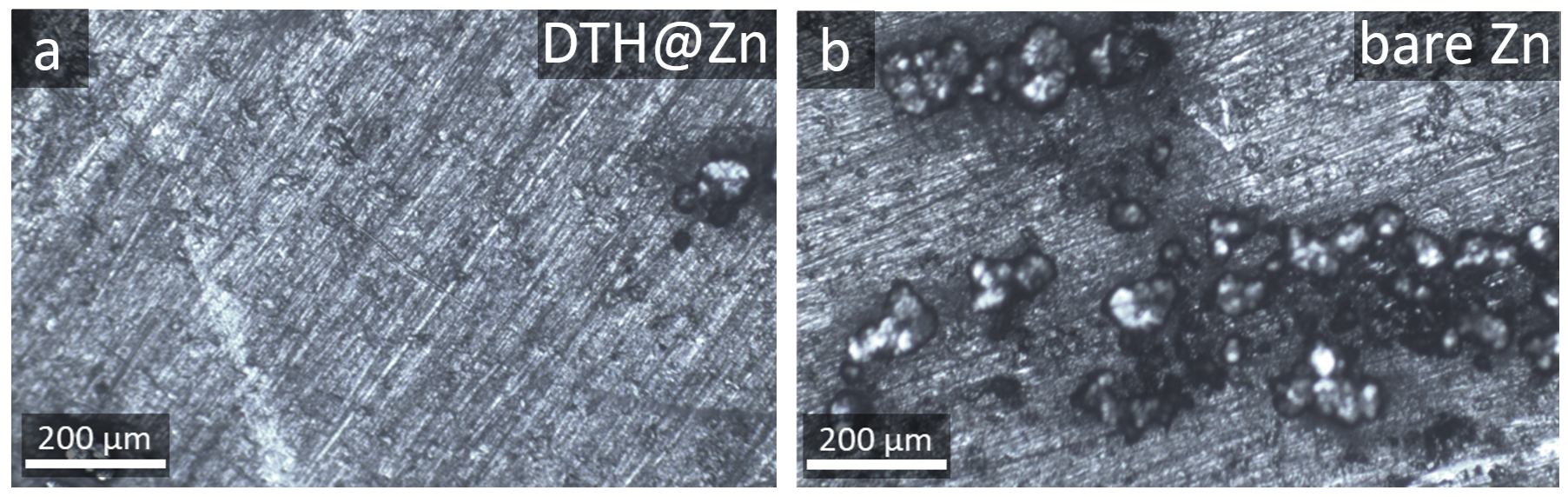


**Supplementary Fig. 13** Confocal optical microscopy images showing the presence of zinc dendrites and byproducts of cycled Bare Zn.


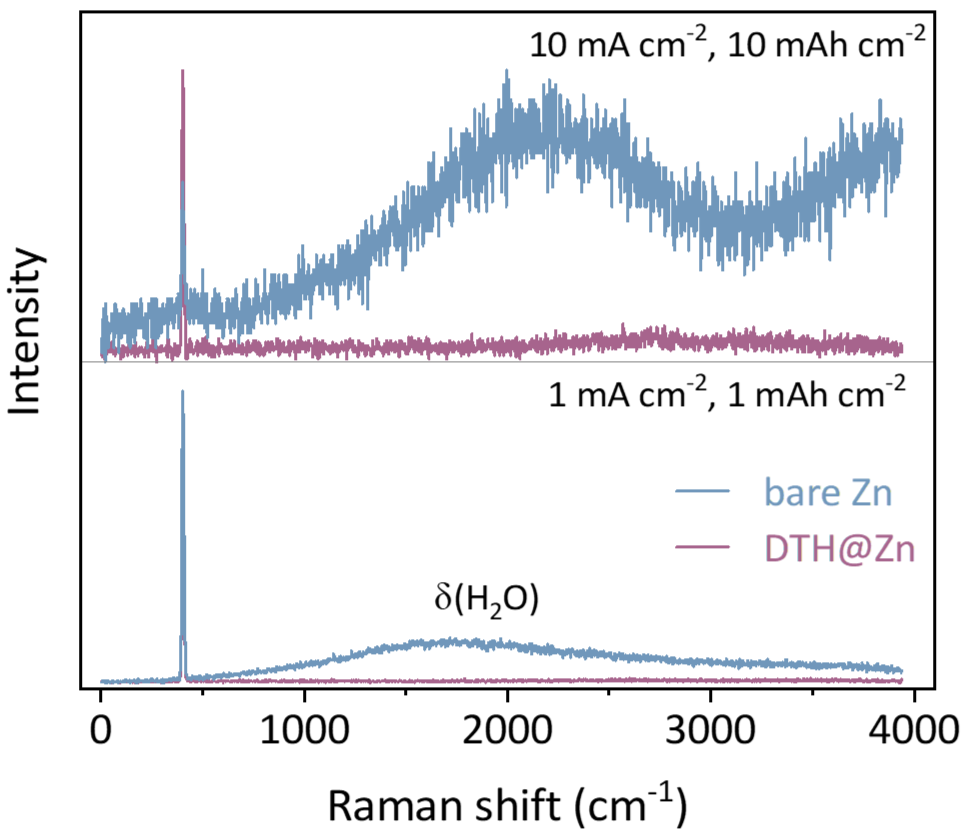


**Supplementary Fig. 14** The Raman spectra of bare Zn and DTH@Zn cycled under different conditions.


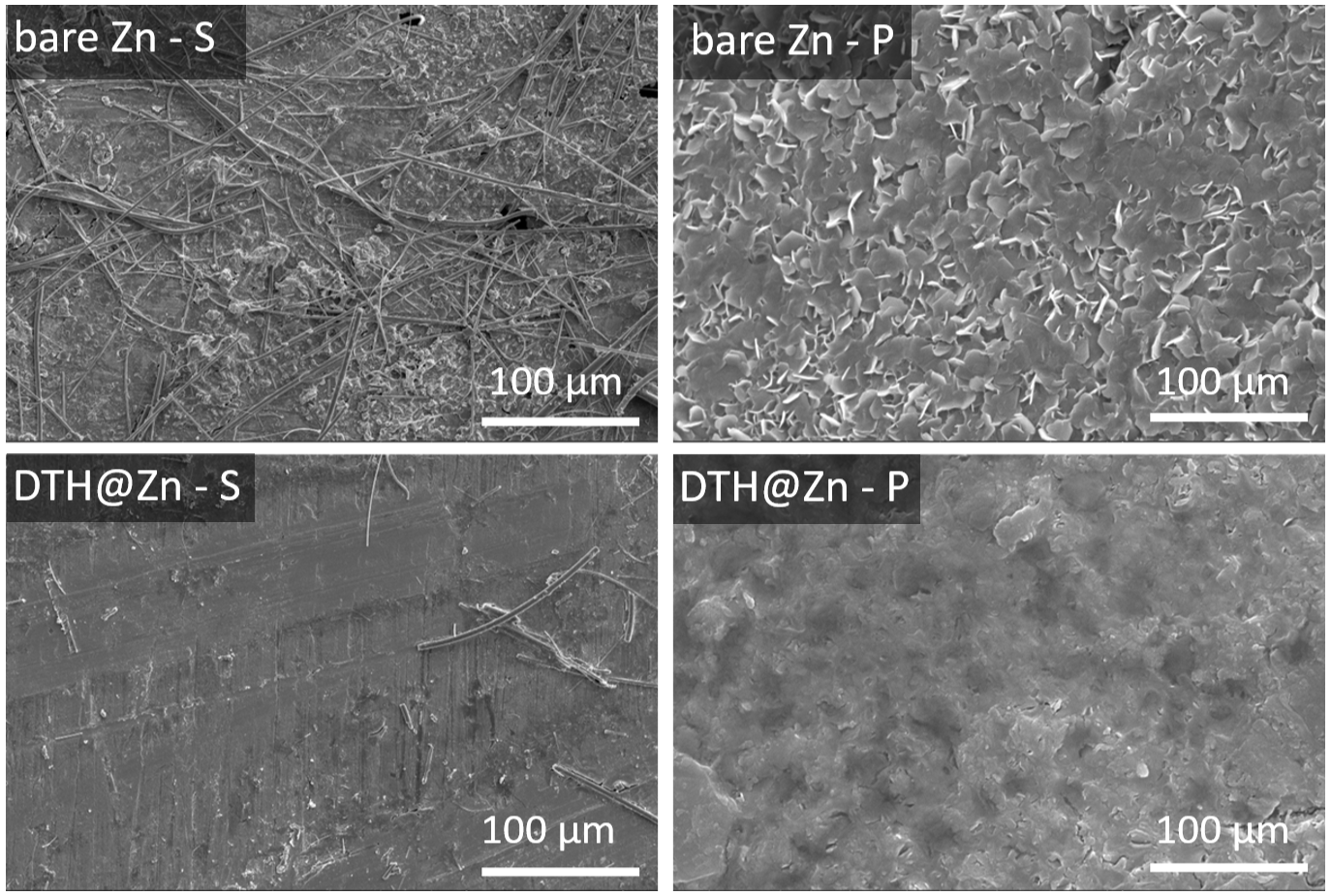


**Supplementary Fig. 15** SEM images of the striped and plated sides of cycled zinc electrodes.


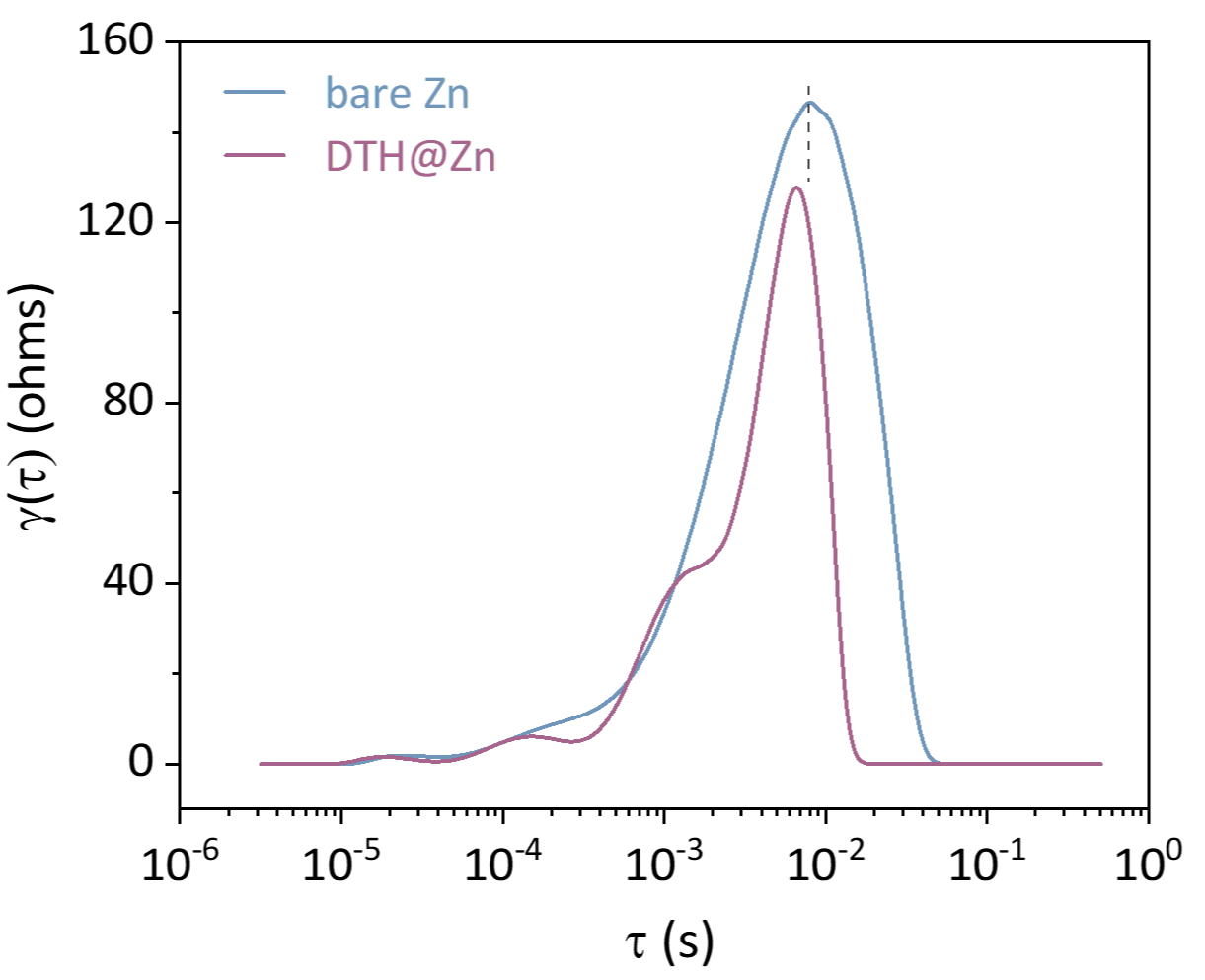


**Supplementary Fig. 16** The distribution of relaxation time (DRT) analysis of bare Zn||Ti and DTH@Zn||Ti cells.


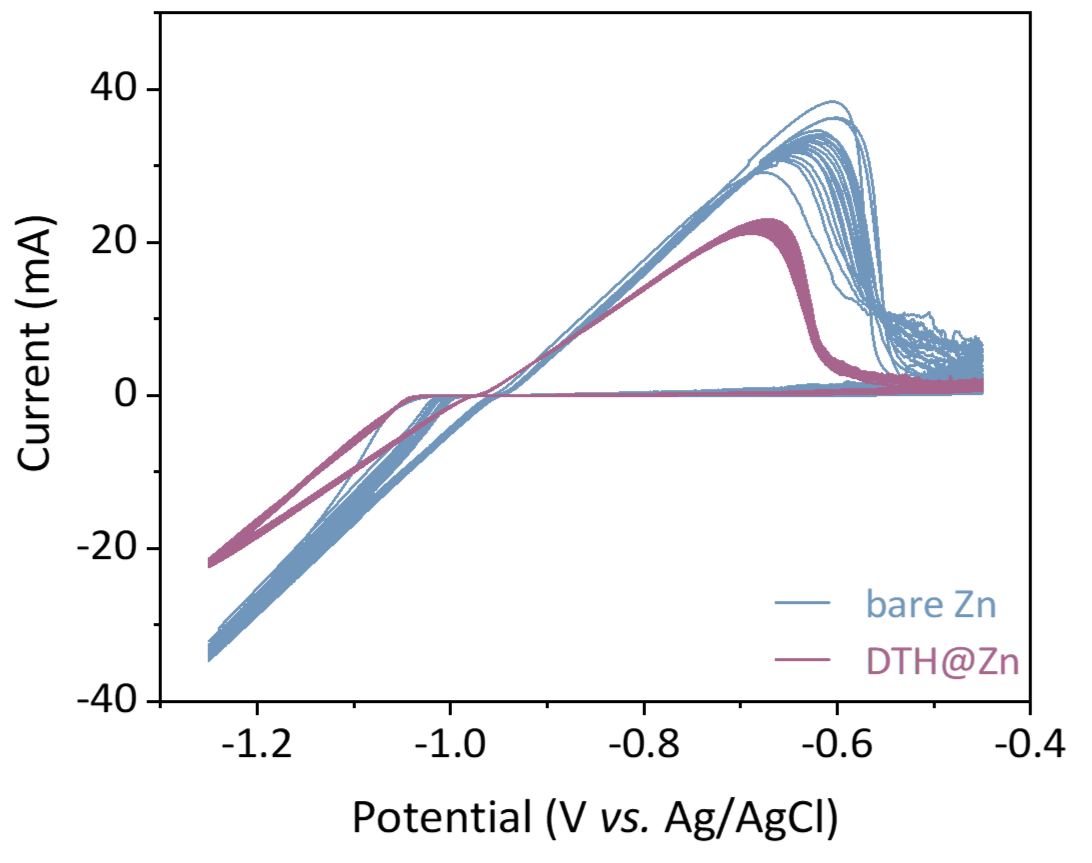


**Supplementary Fig. 17** CV curves of bare Zn||Ti and DTH@Zn||Ti cells scanned at 5 V s^-1^ for 50 cycles.


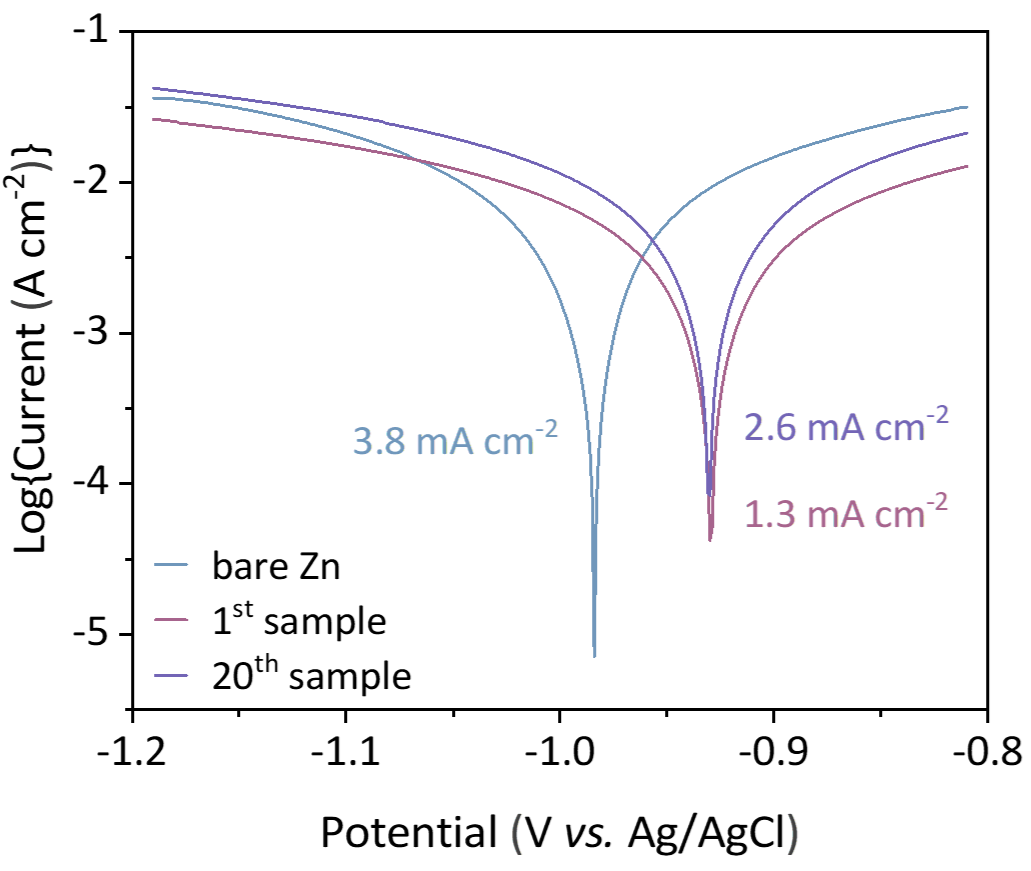


**Supplementary Fig. 18** Linear polarization curves of bare Zn||Ti and DTH@Zn||Ti cells.


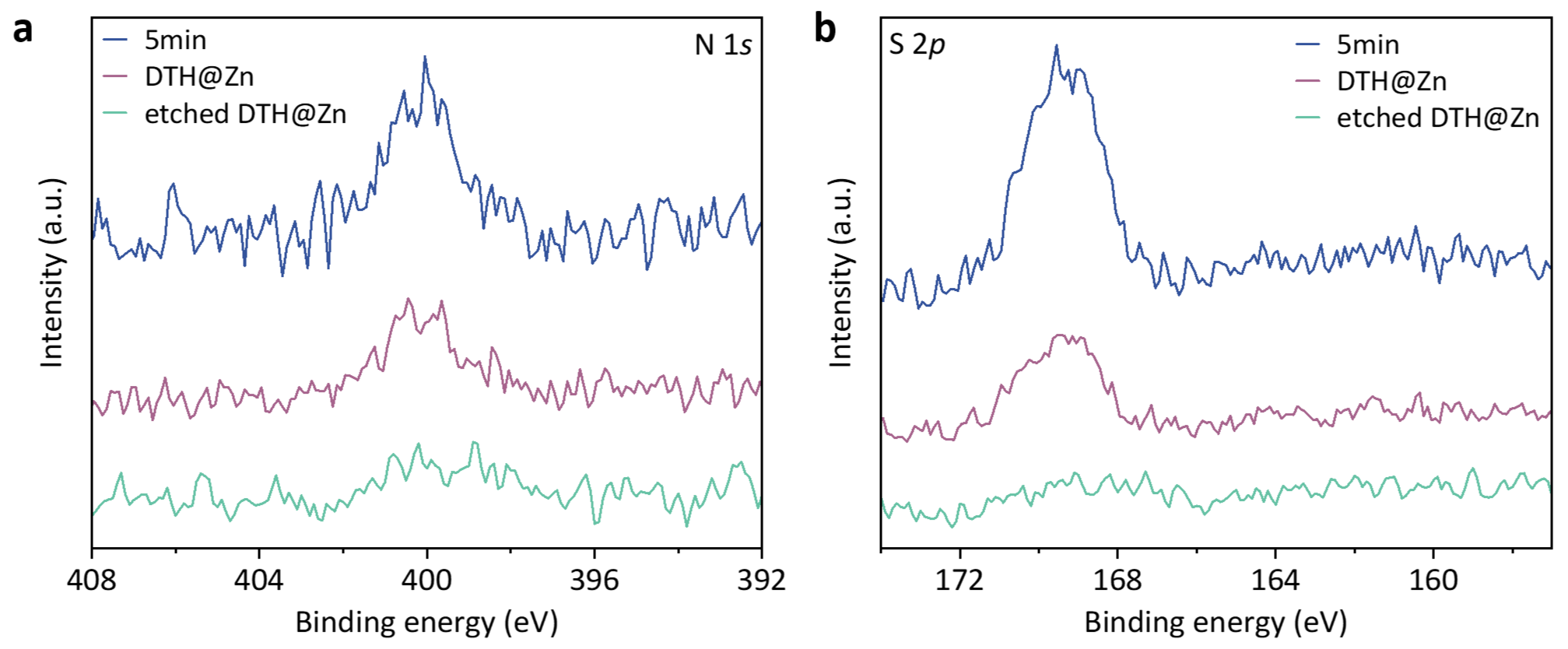


**Supplementary Fig. 19** **a** N 1*s* and **b** S 2*p* core-level XPS spectra of the zinc plate which experienced 5 s (violet curve) and 5 min (blue) of DTH treatment, as well as the one after 30 s etching process (green).


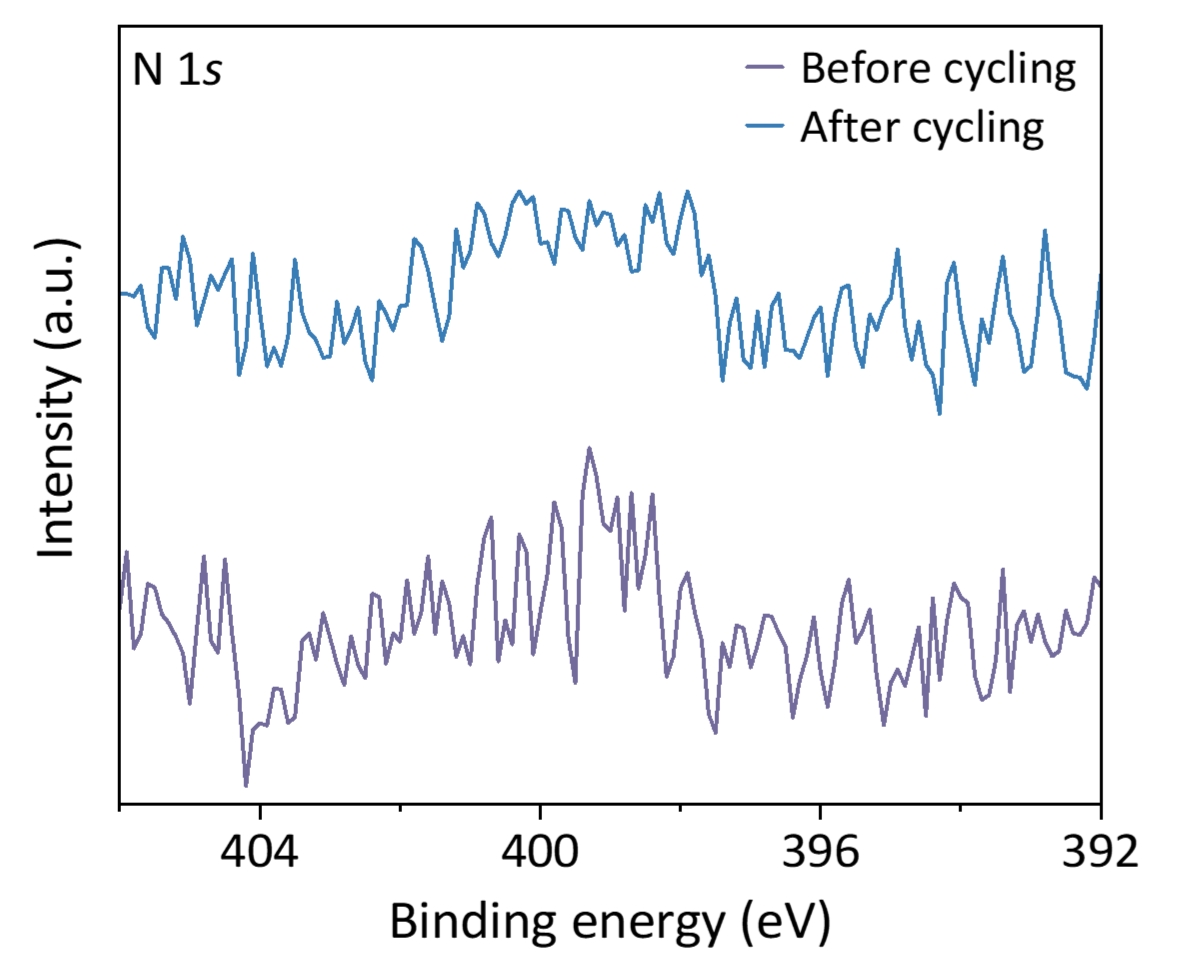


**Supplementary Fig. 20** N1s core-level XPS spectra of DTH@Zn electrode before and after cycling.


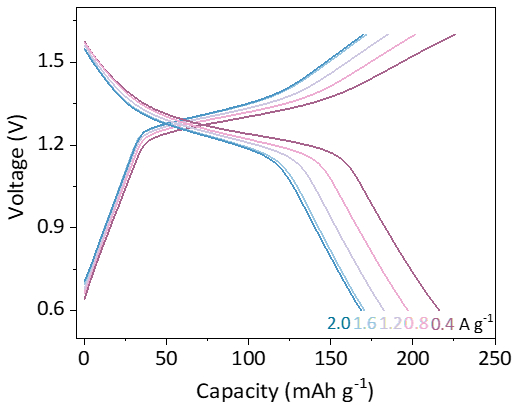


**Supplementary Fig. 21** GCD curves of DTH@Zn||ODASnI_4_ batteries recorded at different current density.


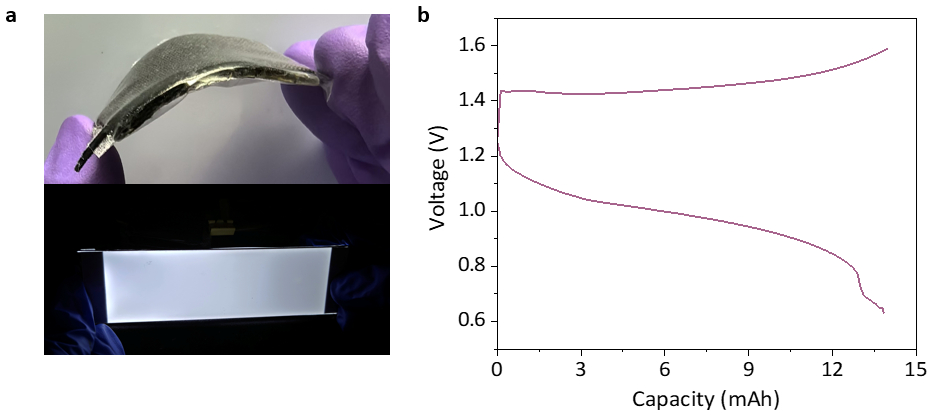


**Supplementary Fig. 22**. (a) the photograph of flexible DTH@Zn||ODASnI_4_ battery and the powered cold light panel; (b) the GCD curve of the flexible battery.

**References**

1. Abdulla, J. et al. Elimination of Zinc Dendrites by Graphene Oxide Electrolyte Additive for Zinc-Ion Batteries. *ACS Appl. Energy Mater.* **4**, 4602-4609 (2021).

2. Qiu, M., Sun, P., Cui, G. & Mai, W. Chaotropic Polymer Additive with Ion Transport Tunnel Enable Dendrite-Free Zinc Battery. *ACS Appl. Mater. Interfaces* **14**, 40951-40958 (2022).

3. Bayaguud, A., Luo, X., Fu, Y. & Zhu, C. Cationic Surfactant-Type Electrolyte Additive Enables Three-Dimensional Dendrite-Free Zinc Anode for Stable Zinc-Ion Batteries. *ACS Energy Lett.* **5**, 3012-3020 (2020).

4. Guo, X. et al. Alleviation of Dendrite Formation on Zinc Anodes via Electrolyte Additives. *ACS Energy Lett.* **6**, 395-403 (2021).

5. Wei, T. et al. Addition of Dioxane in Electrolyte Promotes (002)-Textured Zinc Growth and Suppressed Side Reactions in Zinc-Ion Batteries. *ACS Nano* **17**, 3765-3775 (2023).

6. Huang, H. et al. Boosting Reversibility and Stability of Zn Anodes via Manipulation of Electrolyte Structure and Interface with Addition of Trace Organic Molecules. *Adv. Energy Mater.* **12**, 2202419 (2022).

7. Meng, Q. et al. Attenuating Water Activity Through Impeded Proton Transfer Resulting from Hydrogen Bond Enhancement Effect for Fast and Ultra-Stable Zn Metal Anode. *Adv. Energy Mater.* **13**, 2302828 (2023).

8. Lu, H. et al. Amino Acid‐Induced Interface Charge Engineering Enables Highly Reversible Zn Anode. *Adv. Funct. Mater.* **31**, 2103514 (2021).

9. Wang, H. et al. Stabilizing Zn Anode Interface by Simultaneously Manipulating the Thermodynamics of Zn Nucleation and Overpotential of Hydrogen Evolution. *Adv. Funct. Mater.* **32**, 2207898 (2022).

10. Yang, J. et al. Three Birds with One Stone: Tetramethylurea as Electrolyte Additive for Highly Reversible Zn-Metal Anode. *Adv. Funct. Mater.* **32**, 2209642 (2022).

11. Yang, F. et al. Understanding H_2_ Evolution Electrochemistry to Minimize Solvated Water Impact on Zinc-Anode Performance. *Adv. Mater.* **34**, e2206754 (2022).

12. Liu, D.S. et al. Manipulating OH^-^-Mediated Anode-Cathode Cross-Communication Toward Long-Life Aqueous Zinc-Vanadium Batteries. *Angew Chem. Int. Ed.* **62**, e202215385 (2022).

13. Shi, M. et al. Molecule Engineering of Sugar Derivatives as Electrolyte Additives for Deep-Reversible Zn Metal Anode. *Angew Chem. Int. Ed.* **63**, e202407261 (2024).

14. Cao, L. et al. Solvation Structure Design for Aqueous Zn Metal Batteries. *J. Am. Chem. Soc.* **142**, 21404-21409 (2020).

15. Zhao, K. et al. Boosting the Kinetics and Stability of Zn Anodes in Aqueous Electrolytes with Supramolecular Cyclodextrin Additives. *J. Am. Chem. Soc.* **144**, 11129-11137 (2022).

16. Li, M. et al. Surface Protection and Interface Regulation for Zn Anode via 1-Hydroxy Ethylidene-1,1-Diphosphonic Acid Electrolyte Additive toward High-Performance Aqueous Batteries. *Small* **18**, e2107398 (2022).

17. Wu, F. et al. Achieving Highly Reversible Zinc Anodes via N, N-Dimethylacetamide Enabled Zn-Ion Solvation Regulation. *Small* **18**, e2202363 (2022).
